# Behavioral and psychosocial indicators for depression and anxiety risk stratification among college students: development and external validation of a machine-learning-assisted framework

**DOI:** 10.3389/fpsyg.2026.1972318

**Published:** 2026-10-27

**Authors:** Kailin Mao, Yuqi He, Xiaojian Yin, Lihan Lin, Yikun Zheng, Guopeng Hu

**Affiliations:** 1 School of Physical Education, Huaqiao University, Quanzhou, China; 2 Shanghai Institute of Technology, Shanghai, China; 3 Provincial University Key Laboratory of Sport and Health Science, School of Physical Education and Sport Science, Fujian Normal University, Fuzhou, China

**Keywords:** anxiety, behavioral indicators, college students, depression, psychosocial factors, risk stratification

## Abstract

**Background:**

Depression and anxiety symptoms are common among college students. Behavioral and psychosocial indicators may support preventive campus mental health work, but risk-stratification frameworks require external validation.

**Methods:**

We developed a construct-level model-explanation framework using questionnaire data from a development sample. The cleaned dataset included 1,267 students; internal-validation analyses included 1,096 students for depression risk and 1,095 for anxiety risk. Risk was defined as a Patient Health Questionnaire-9 (PHQ-9) or Generalized Anxiety Disorder-7 (GAD-7) score of 10 or higher. The primary Feature set C random-forest (RF) pipelines integrated demographic, traditional, and emerging behavioral and psychosocial indicators. Fold-specific preprocessing and percentile cutoffs were used for internal validation. Locked pipelines were applied without retraining, predictor reselection, optimization, recalibration, or threshold adjustment to an independent university sample of 1,468 students.

**Results:**

External area under the receiver operating characteristic curve (AUROC), area under the precision–recall curve (PR-AUC), and Brier scores were 0.813 (95% CI, 0.776–0.848), 0.402 (0.325–0.491), and 0.090 (0.083–0.098), respectively, for depression and 0.809 (0.765–0.851), 0.205 (0.144–0.300), and 0.063 (0.056–0.070), respectively, for anxiety. Ordinary participant-level bootstrap was the primary approach, with stratified bootstrap as a sensitivity analysis. Depression calibration was close to the prevalence baseline (Brier 0.091; intercept −0.668; slope 1.401), whereas anxiety showed miscalibration (Brier 0.063 vs. baseline 0.051; intercept −1.227; slope 1.102). The models supported external discrimination and risk ranking but did not validate individual absolute-risk probabilities or clinical thresholds.

**Conclusion:**

Behavioral and psychosocial indicators supported externally validated risk stratification in an independent university sample. Anxiety-risk findings should be interpreted as preliminary because the number of positive cases was limited. Further evaluation is needed before wider implementation.

## Background

Depression and anxiety symptoms among college students are important public health and educational concerns. University life is accompanied by academic demands, social transitions, irregular schedules, digital technology use, and changing health behaviors. Large international student mental health surveys and recent meta-analytic evidence have shown that common mental health symptoms are frequent in college populations, yet many students do not receive timely support (Auerbach et al., 2018; Lipson et al., 2022; Li et al., 2022). Scalable methods for identifying students at elevated risk may therefore have value for prevention, triage, and campus health promotion, provided that such methods are reported cautiously and externally validated before implementation.

The majority of questionnaire-based studies of student mental health have evaluated individual exposures or small groups of risk indicators. Traditional health behaviors, such as physical activity, sedentary behavior, sleep duration, smoking, alcohol consumption, and dietary habits, remain relevant to mental health risk. The behavioral environment of contemporary college students also includes smartphone engagement, recreational screen time, social jetlag, body-image concern, and psychosocial resources such as perceived social support. Evidence links perceived social support with student mental health (Hefner and Eisenberg, 2009; Ruihua et al., 2025; Vicary et al., 2025), problematic smartphone use with mental health and sleep-related outcomes (Li et al., 2020; Sohn et al., 2019), sleep timing misalignment with depressive symptoms (Wittmann et al., 2006; Luo et al., 2025), body dissatisfaction with mental health (Paxton et al., 2006), and breakfast and dietary-rhythm behaviors with mental health symptoms (Wang et al., 2025). These indicators may collectively provide predictive information beyond demographic and traditional lifestyle variables.

Risk stratification in student mental health requires approaches that integrate heterogeneous behavioral and psychosocial indicators while remaining transparent about uncertainty and calibration. Machine-learning (ML) models can serve this analytic role, but methodological rigor is essential. Apparent accuracy can be misleading when outcomes are imbalanced. Transparent reporting under the Transparent Reporting of a multivariable prediction model for Individual Prognosis or Diagnosis with artificial intelligence extensions (TRIPOD+AI) and applicability assessment under the Prediction model Risk Of Bias Assessment Tool with artificial intelligence extensions (PROBAST+AI) are relevant to prediction model research (Collins et al., 2024; Moons et al., 2025). Models that rank individuals well may still provide poorly calibrated probabilities, so discrimination and calibration should be reported together (Steyerberg, 2019; Van Calster et al., 2019). Precision–recall metrics are particularly informative for low-prevalence binary outcomes (Saito and Rehmsmeier, 2015; Davis and Goadrich, 2006). Recent student-focused prediction studies and systematic reviews further emphasize algorithm comparison and the reporting of multiple performance metrics (Schaab et al., 2024; Zhai et al., 2025; El Morr et al., 2024; Meda et al., 2023; Shin et al., 2025).

External validation is essential for evaluating whether a risk-stratification framework developed in one university sample retains performance in an independent student population (Riley et al., 2024). It provides preliminary evidence of transportability (Altman and Royston, 2000), although it does not by itself establish broad transportability.

This study developed and internally validated a behavioral and psychosocial risk-stratification framework with construct-level model explanation for depression and anxiety risk among college students. Machine-learning models were used as analytical tools to integrate multidimensional indicators; the primary contribution was the externally validated framework rather than a new algorithm. The analysis compared demographic, traditional health-behavior, and emerging health-risk/psychosocial feature sets and examined calibration, threshold-dependent performance, permutation-based model explanation, and sensitivity analyses.

## Methods

### Study design and participants

This cross-sectional model-development and external-validation study used questionnaire data from two independent university samples. The development sample was obtained from a single university, and the Shanghai external-validation cohort provided through the Shanghai Institute of Technology was treated as an independent external sample. Development questionnaire submissions were recorded from 25 November 2025 to 4 May 2026. Exact external recruitment dates could not be reconstructed from the retained study records. The raw development file contained 1,392 records, of which 1,385 included recorded consent.

The analysis followed current recommendations for transparent prediction model development and validation, including predefined outcome definitions, feature-set construction, internal validation, calibration assessment, and sensitivity analyses (Collins et al., 2024; Moons et al., 2025).

### Eligibility and data-quality criteria

Development records were retained in the cleaned dataset if consent was recorded and the submission remained after duplicate review. Potential duplicates were identified using name, student identifier, sex, college, and major when available; within suspected duplicate groups, records with greater completion were retained, followed by greater data completeness and later submission time. Excluding 118 duplicate records yielded 1,267 students. Outcome-specific analyses additionally required a calculable PHQ-9 or GAD-7 total. Implausible predictor values were set to missing rather than used as record-level exclusion criteria. Figure 1 summarizes sample eligibility and the modeling pathway.

**Figure 1:**
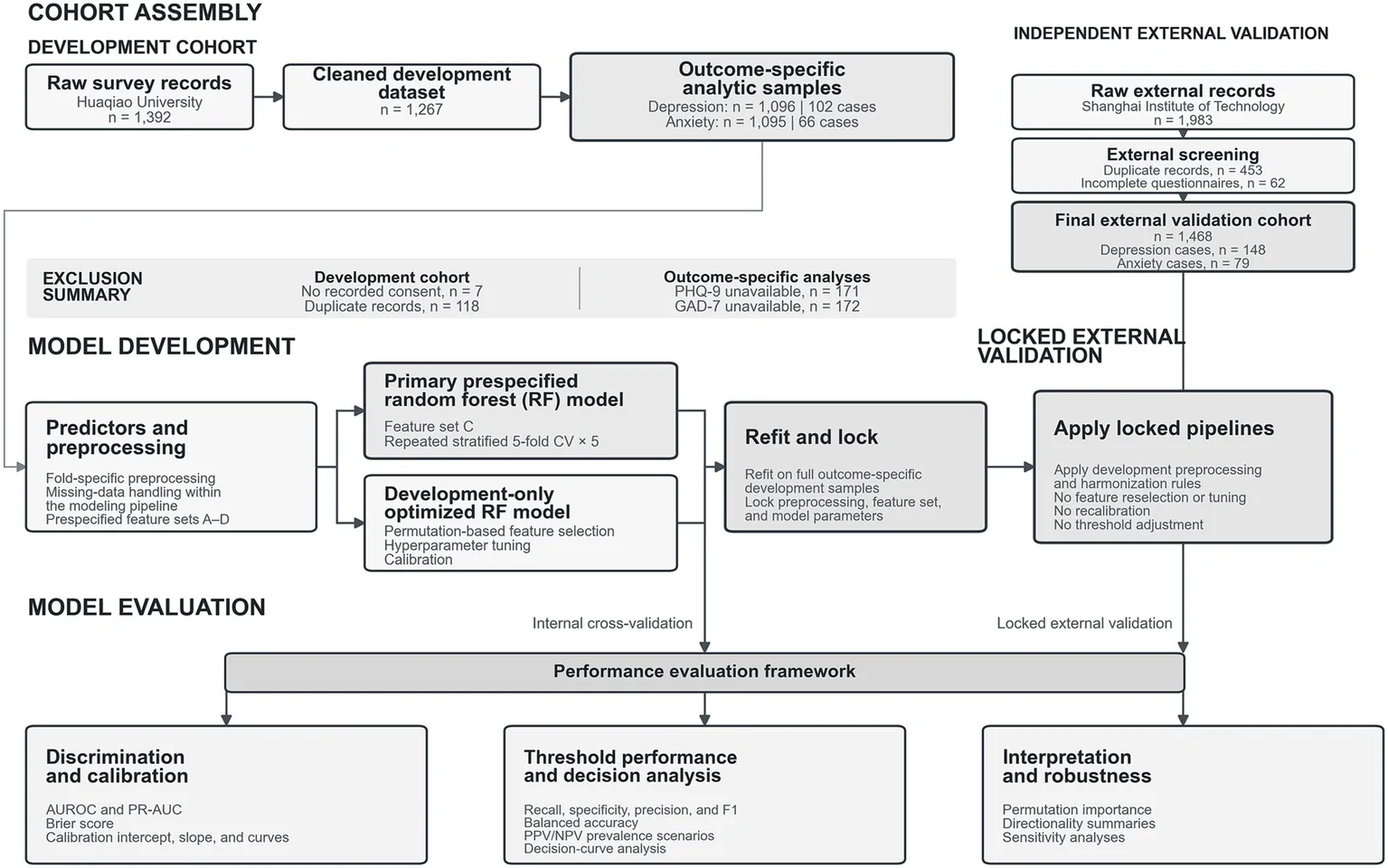
Study flow and modeling pathway. The development sample progressed through consent and duplicate review to a cleaned dataset and parallel depression-risk and anxiety-risk analyses, repeated internal validation, and refitting of the final Feature set C random-forest pipelines. The external sample progressed through duplicate review and completeness screening, predictor harmonization, and application of the saved pipelines without retraining. Outcome-specific analyses remained parallel throughout. The development-only optimization pathway is shown for historical traceability; its pre-correction internal-validation estimates are not treated as formal corrected results.

### External-validation sample

External-validation data were obtained from the Shanghai cohort provided by the Shanghai Institute of Technology. The external file contained 1,983 records; 1,530 remained after duplicate review, and the 1,468 records marked as complete in the questionnaire system formed the external-validation sample. Both outcome analyses included 1,468 students, with 148 depression-risk cases (10.1%) and 79 anxiety-risk cases (5.4%). Potential duplicates were identified using student identifier, name, sex, and date of birth when available; complete records, records with greater data completeness, and later records or submission times were retained in that order. The process excluded 453 duplicate records from 336 suspected groups involving 789 records before completeness screening (Supplementary Table S1). Personal identifiers were excluded from the external-validation analysis files. The external sport field was mapped to sports_major using the existing binary rule (sport = 1 as sports-major and other non-missing values as non-sports-major). The data provider confirmed that sport = 2 denotes a non-sports major; all retained Shanghai records had sport = 2, so sports_major = 0 for all records. The 62 records excluded at the completeness stage were not marked complete in the questionnaire system. Duplicate records were resolved by retaining one record per suspected group according to the stated priority, without field-level reconciliation across duplicate records.

### Assessment of depression and anxiety risk

Depression symptoms were assessed using the nine PHQ-9 items, with each item scored from 0 to 3 and total scores ranging from 0 to 27 (Kroenke et al., 2001). Depression risk was defined as a PHQ-9 total score of 10 or higher. Anxiety symptoms were assessed using the seven GAD-7 items, with total scores ranging from 0 to 21 (Spitzer et al., 2006). Anxiety risk was defined as a GAD-7 total score of 10 or higher. Participants with incomplete item information that prevented calculation of the corresponding PHQ-9 or GAD-7 total score were excluded from that outcome-specific analysis. These thresholds were used to identify students with at least moderate symptom severity and should not be interpreted as clinical diagnoses. PHQ-9 and GAD-7 items, total scores, and variables derived directly from self-harm or suicidal-behavior questions were excluded from all predictor sets to avoid outcome leakage.

### Predictor variables and feature sets

Candidate predictors covered demographic, anthropometric, lifestyle, digital-behavior, sleep-rhythm, dietary, body-image, social-support, and general health-status domains. Constructs were selected *a priori* based on public health relevance and literature linking perceived social support (Hefner and Eisenberg, 2009), digital engagement (Li et al., 2020; Sohn et al., 2019), sleep and circadian timing (Wittmann et al., 2006; Luo et al., 2025), body image (Paxton et al., 2006), dietary habits (Wang et al., 2025), and physical activity and sedentary behavior (World Health Organization, 2020) with student mental health. Feature set A included demographic and basic socioeconomic variables. Feature set B added traditional health behaviors. Feature set C added emerging behavioral and psychosocial indicators and was selected as the primary construct-level feature set. Feature set D contained more granular and correlated variables and was retained as a sensitivity model. Operational definitions, feature-set membership, measurement characteristics of questionnaire-derived scores, and patterns of missingness are reported in Supplementary Tables S2a–S2c and S3. Social support was included because lower perceived support has been associated with poorer mental health outcomes among university students (Hefner and Eisenberg, 2009).

Problematic smartphone use has been associated with depression and anxiety symptoms (Li et al., 2020; Sohn et al., 2019). It has also been associated with sleep-related problems (Crowhurst and Hosseinzadeh, 2024; Yin et al., 2025). Sleep timing and social jetlag indicators were incorporated because circadian misalignment has been associated with depressive symptoms in college populations (Wittmann et al., 2006; Luo et al., 2025). Body-image indicators were included based on evidence linking body dissatisfaction to mental health outcomes (Paxton et al., 2006). Dietary-rhythm indicators were considered based on evidence regarding breakfast habits and mental health symptoms among students (Wang et al., 2025). Physical activity and sedentary behavior domains were retained according to international public health recommendations (World Health Organization, 2020).

Some continuous questionnaire-derived scores and their dichotomized high-risk indicators represented related constructs. The indicators were retained in Feature set C to represent prespecified threshold-based information, while the continuous scores preserved gradient information. Predictor importance was therefore interpreted at the construct level rather than as independent information from every listed variable.

### Model development, feature selection, and internal validation

The primary benchmark compared logistic regression, random forest, and support vector machine (SVM) classifiers for Feature set C (Supplementary Table S4). Model specifications are reported in Supplementary Table S5. The primary benchmark retained the prespecified Feature set C predictors; the random forest was selected after algorithm-level comparison for its combination of discrimination profile and support for descriptive model-level explanation (Schaab et al., 2024; Zhai et al., 2025; El Morr et al., 2024; Meda et al., 2023; Shin et al., 2025; Breiman, 2001). Random forest remains a complex ensemble model, and the current analysis does not provide individual-level or clinical explanations. Numeric predictors were median-imputed and standardized within each training fold, whereas categorical predictors were imputed with the most frequent category and one-hot encoded. All preprocessing was estimated within the training fold and then applied to the held-out fold. Analyses used scikit-learn (Pedregosa et al., 2011). Analyses were performed in Python 3.13.5 using scikit-learn 1.6.1 (Pedregosa et al., 2011), pandas 2.2.3, NumPy 2.1.3, SciPy 1.15.3, and Matplotlib 3.10.0. The overall analysis workflow is shown in Supplementary Figure S1. Missing values were handled within this fold-specific modeling pipeline rather than by complete-case deletion as the primary analysis.

Internal validation used repeated stratified 5-fold cross-validation (CV) with 5 repeats, yielding 25 validation folds. Percentile-derived binary indicators were recalculated within each training fold and applied to the held-out fold. Model performance was summarized using pooled participant-level AUROC, average precision (reported as PR-AUC), Brier score, recall, specificity, precision, F1 score, and balanced accuracy (Steyerberg, 2019; Brier, 1950). Default-threshold classification metrics used a threshold of 0.5. The development-derived Youden thresholds (Youden, 1950) were retained for transparent threshold evaluation. Because the outcomes were imbalanced, PR-AUC was reported as a complementary measure to AUROC (Saito and Rehmsmeier, 2015; Davis and Goadrich, 2006), and calibration was assessed separately (Van Calster et al., 2019).

The archived development-only optimization pathway was retained for traceability; after correction of the preprocessing workflow, its estimates were not treated as formally corrected internal-validation results.

### Calibration, interpretation, and sensitivity analyses

Internal calibration was examined using pooled participant-level predictions from the corrected fold-specific pipeline. The corrected Feature set C calibration intercept/slope values were −0.891/1.119 for depression risk and −0.961/1.129 for anxiety risk. The earlier sigmoid and isotonic calibration comparisons were generated before the preprocessing correction and are not retained as formal corrected estimates. These results characterize internal calibration of the corrected pipeline and do not establish externally validated individual risk probabilities.

Permutation importance based on AUROC was used as a descriptive model-level explanation. Directionality summaries compared observed event rates and mean predicted probabilities across quartiles or categories of higher-ranked variables (Molnar, 2022). For the external locked models, related variables were jointly permuted within eight prespecified constructs; group importance was defined as the decrease in AUROC relative to the baseline external prediction, with results reported separately in Supplementary Table S21. These analyses characterize model behavior and do not establish causal effects.

### External-validation analysis

After corrected internal validation, the final Feature set C random-forest pipelines were refitted on the full outcome-specific development samples and then applied to the external-validation dataset without retraining, predictor reselection, hyperparameter optimization, recalibration, or threshold adjustment. The external cohort was used only for final model evaluation. Feature engineering followed the development-sample rules. Sleep-time variables in the external-validation dataset were converted from seconds since midnight to clock-time values before deriving weighted sleep duration, short sleep, social jetlag, and high social jetlag according to the development-sample rules. Four distribution-based binary indicators used the fixed development-sample cutoffs. All sleep-time conversion and derived-variable construction were performed before and independently of outcome-specific analyses and model fitting; this preprocessing was outcome-blind. External-validation metrics included AUROC, PR-AUC, Brier score, calibration intercept, calibration slope, recall, specificity, precision, F1 score, and balanced accuracy. Ordinary participant-level bootstrap was the primary external uncertainty analysis; stratified bootstrap was a sensitivity analysis. The fixed internal Youden thresholds were 0.226 for depression risk and 0.251 for anxiety risk and were not re-estimated in the Shanghai external-validation cohort. For the locked primary Feature set C predictions, decision-curve analysis evaluated thresholds from 0.05 to 0.30 against treat-all and treat-none strategies (Supplementary Figure S5).

For locked external predictions, positive predictive value (PPV) and negative predictive value (NPV) were estimated under assumed prevalence levels of 5, 10, 15, and 20% using external-validation sensitivity and specificity at the fixed development-derived thresholds.

### Sensitivity analyses

Sensitivity analyses included complete-case analysis, removal of sleep and rhythm variables, removal of dichotomized predictors that duplicated continuous scores, removal of anthropometric variables, logistic-regression robustness models, and the full Feature set D random-forest model. For the external-validation sensitivity analysis, one Feature set D pipeline was refit for each outcome on the full outcome-specific development sample using its fixed 70-variable specification and then applied to Shanghai; the results are reported in Supplementary Table S25. Because several archived analyses used legacy materialized percentile indicators, their numerical internal-validation estimates were not treated as formal results after correction of the preprocessing workflow. The corrected Feature set C estimates are reported in Table 1 and Supplementary Table S20. Development-only predictor-selection frequencies are provided in Supplementary Table S23; these are descriptive traceability outputs from the archived development-only selection pathway and are not treated as formal leakage-free model-selection estimates. A prediction-only all-0/all-1 sports_major mapping sensitivity using the locked primary pipelines is provided in Supplementary Table S24; this did not replace the primary external estimates. Because the anxiety outcome had 66 positive development cases and 79 positive external cases, the archived anxiety predictor rankings, number of selected predictors, and tuning outputs were treated as exploratory and potentially unstable.

**Table 1:** Internal-validation performance of random-forest models across feature sets.

Outcome	Feature set	AUROC (corrected pooled CV)	PR-AUC (corrected pooled CV)	Brier score (corrected pooled CV)	Recall	Specificity
Depression risk	A	0.506	0.113	0.123	0.020	0.982
Depression risk	B	0.657	0.147	0.108	0.000	0.981
Depression risk	C	0.773	0.266	0.091	0.176	0.963
Depression risk	D	0.812	0.369	0.081	0.157	0.991
Anxiety risk	A	0.602	0.107	0.084	0.045	0.989
Anxiety risk	B	0.634	0.119	0.075	0.015	0.999
Anxiety risk	C	0.796	0.266	0.062	0.167	0.984
Anxiety risk	D	0.849	0.317	0.055	0.106	0.994

PR-AUC was calculated as average precision. Feature set A contained no percentile-derived indicators; Feature sets B–D used fold-specific percentile cutoffs and training-fold preprocessing.

## Results

### Sample characteristics and outcome availability

The raw questionnaire file contained 1,392 records. After consent screening and duplicate exclusion, the cleaned analytic dataset included 1,267 students. Depression-risk status was available for 1,096 students, of whom 102 were classified as positive cases (9.31%); 171 students lacked complete PHQ-9 outcome data and were excluded from the depression-specific analysis. Anxiety-risk status was available for 1,095 students, of whom 66 were classified as positive cases (6.03%); 172 students lacked complete GAD-7 outcome data and were excluded from the anxiety-specific analysis. The development-sample study flow is summarized in Figure 1. Table 2 summarizes participant characteristics by depression-risk status; anxiety-stratified characteristics are provided in Supplementary Table S8. Comparisons between participants included in and excluded from the outcome-specific analyses because of missing PHQ-9 or GAD-7 outcome data are provided in Supplementary Table S9.

**Table 2:** Participant characteristics by depression-risk status.

Characteristic	Overall	Depression-risk negative	Depression-risk positive	*p*-value
*N*	1,096	994	102	
Age, years, mean ± SD	19.17 ± 1.11	19.14 ± 1.09	19.47 ± 1.26	0.003
BMI, kg/m^2^, mean ± SD	21.97 ± 4.87	21.96 ± 4.83	22.06 ± 5.28	0.955
Waist circumference, cm, mean ± SD	76.78 ± 11.26	76.91 ± 11.26	75.55 ± 11.21	0.294
Weighted sedentary hours, mean ± SD	5.82 ± 3.27	5.82 ± 3.29	5.85 ± 3.06	0.645
Recreational screen time, hours, mean ± SD	4.10 ± 3.24	4.05 ± 3.22	4.54 ± 3.41	0.229
Smartphone dependency score, mean ± SD	24.50 ± 8.88	23.77 ± 8.39	31.65 ± 10.31	<0.001
Weighted sleep duration, hours, mean ± SD	8.12 ± 1.13	8.14 ± 1.10	7.87 ± 1.42	0.181
Social jetlag, hours, mean ± SD	1.38 ± 1.08	1.36 ± 1.08	1.59 ± 1.07	0.043
Body-image concern score, mean ± SD	19.16 ± 9.57	18.53 ± 9.18	25.35 ± 11.03	<0.001
Social support score, mean ± SD	59.32 ± 13.32	59.92 ± 13.24	53.48 ± 12.82	<0.001
Female, *n* (%)	534 (48.7)	477 (48.0)	57 (55.9)	0.157
Sports major, *n* (%)	77 (7.0)	65 (6.5)	12 (11.8)	0.078
Physical inactivity, *n* (%)	220 (20.1)	195 (19.6)	25 (24.5)	0.296
High sedentary time, *n* (%)	321 (29.3)	289 (29.1)	32 (31.4)	0.710
High smartphone dependency, *n* (%)	303 (27.6)	242 (24.3)	61 (59.8)	<0.001
Short sleep, *n* (%)	129 (14.4)	110 (13.4)	19 (26.4)	0.004
Evening chronotype, *n* (%)	655 (59.8)	584 (58.8)	71 (69.6)	0.043
Breakfast skipping, *n* (%)	465 (42.4)	409 (41.1)	56 (54.9)	0.010
Current smoking status, *n* (%)	39 (3.6)	29 (2.9)	10 (9.8)	0.002
Current alcohol consumption, *n* (%)	216 (19.7)	185 (18.6)	31 (30.4)	0.007
High body-image concern, *n* (%)	284 (25.9)	232 (23.3)	52 (51.0)	<0.001
Low social support, *n* (%)	289 (26.4)	241 (24.2)	48 (47.1)	<0.001

Depression risk was defined as PHQ-9 ≥ 10. Values are shown as mean ± SD or *n* (%). *p*-values were obtained using the Welch *t*-tests or Mann–Whitney U tests for continuous variables and chi-squared tests or Fisher’s exact tests for categorical variables, as appropriate. Selected characteristics are shown to describe the analytic sample; full anxiety-stratified results are provided in Supplementary Table S8. Percentages were calculated using non-missing denominators; denominators may vary across variables because of missing data.

### Internal-validation performance

The corrected pooled cross-validated estimates showed limited discrimination for the demographic-only model, with incremental improvement after adding traditional health-behavior and emerging behavioral/psychosocial variables. For depression risk, Feature sets A–D achieved AUROC values of 0.506, 0.657, 0.773, and 0.812, with PR-AUC values of 0.113, 0.147, 0.266, and 0.369, respectively. For anxiety risk, the corresponding AUROC values were 0.602, 0.634, 0.796, and 0.849, with PR-AUC values of 0.107, 0.119, 0.266, and 0.317. Feature set A contained no percentile-derived indicators; for Feature sets B-D, percentile cutoffs were estimated within each training fold. The corrected pooled estimates are reported in Table 1 and illustrated in Figure 2. The earlier nested optimization tables are retained only as traceability artifacts and are not formal internal-validation results.

**Figure 2:**
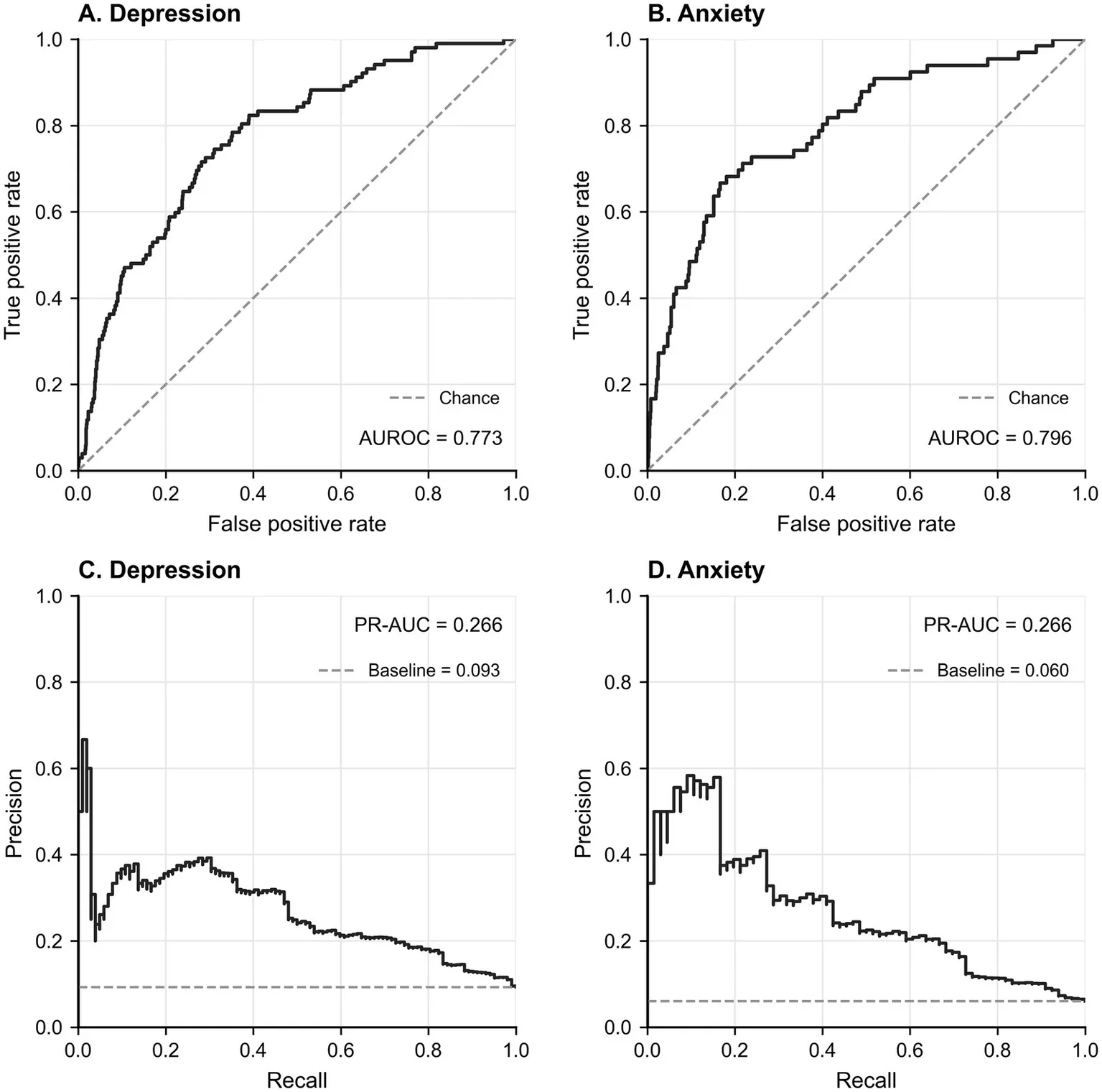
Internal-validation ROC and precision-recall curves for the primary Feature set C random-forest models. **(A,B)** ROC curves for depression risk and anxiety risk, respectively; **(C,D)** precision-recall curves for depression risk and anxiety risk, respectively. Curves are based on participant-level mean out-of-fold probabilities from repeated stratified five-fold cross-validation with five repeats (depression, n = 1,096; anxiety, n = 1,095). PR-AUC was calculated as average precision. Dashed diagonal lines indicate chance discrimination, and dashed horizontal lines indicate outcome prevalence.

### Calibration and threshold-dependent performance

At the default threshold of 0.5, the corrected Feature set C models showed high specificity but limited recall. For depression risk, default-threshold recall and specificity were 0.176 and 0.963; for anxiety risk, they were 0.167 and 0.984. At the fixed development-derived thresholds of 0.226 and 0.251, recall/specificity were 0.716/0.710 for depression risk and 0.636/0.845 for anxiety risk. Corrected calibration intercepts/slopes were −0.891/1.119 for depression risk and −0.961/1.129 for anxiety risk. Formal corrected estimates are summarized in Table 3, with the corresponding internal calibration curves shown in Figure 3; pre-correction sigmoid/isotonic Brier comparisons were not retained as formal results.

**Figure 3:**
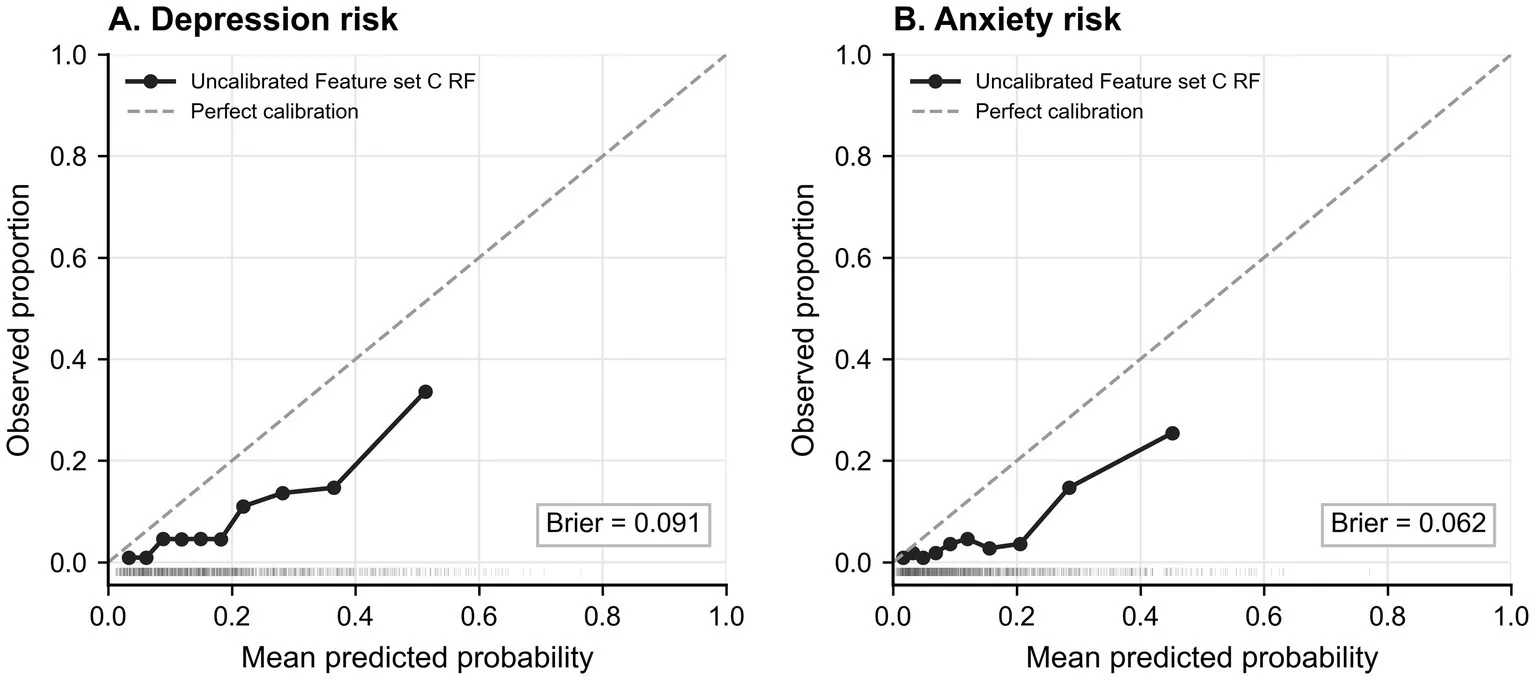
Observed calibration of the uncalibrated primary Feature set C random-forest models for **(A)** depression risk and **(B)** anxiety risk. Calibration curves based on ten quantile bins show the agreement between mean predicted probability and observed event fraction, using participant-level mean out-of-fold probabilities from repeated stratified five-fold cross-validation with five repeats (depression, n = 1,096; anxiety, n = 1,095). Dashed diagonal lines indicate perfect calibration, and prediction rugs below the curves show the empirical distribution of individual predicted probabilities. Both panels display the full 0–1 probability range. These curves assess internal probability calibration and do not establish externally validated individual risk estimates.

**Table 3:** Calibration and threshold analysis for the primary Feature set C random-forest models.

Metric	Depression risk	Anxiety risk
Outcome sample size, n	1,096	1,095
Observed event rate	0.093	0.060
AUROC (corrected pooled CV)	0.773	0.796
PR-AUC / average precision (corrected pooled CV)	0.266	0.266
Uncalibrated Brier score (corrected pooled CV)	0.091	0.062
Baseline Brier score	0.084	0.057
Calibration intercept (corrected pooled CV)	−0.891	−0.961
Calibration slope (corrected pooled CV)	1.119	1.129
Sigmoid-calibrated Brier score	Not retained	Not retained
Isotonic-calibrated Brier score	Not retained	Not retained
Default-threshold recall	0.176	0.167
Default-threshold specificity	0.963	0.984
Fixed development-derived Youden threshold	0.226	0.251
Fixed-threshold recall	0.716	0.636
Fixed-threshold specificity	0.710	0.845

Metrics are pooled participant-level estimates from repeated stratified 5-fold cross-validation with fold-specific percentile cutoffs and training-fold preprocessing. Pre-correction sigmoid and isotonic Brier comparisons were not retained as formal corrected estimates. Fixed threshold rows use the original development-derived thresholds for transparent evaluation.

### Important predictors and directionality

Table 4 summarizes each construct by summing the variable-level permutation-importance values of its prespecified continuous and dichotomized representations. Because related representations can share information, the external joint group-permutation analysis is reported separately in Supplementary Table S21.

**Table 4:** Construct-level predictor importance for the primary Feature set C random-forest models.

Rank	Depression risk construct	Importance	Anxiety risk construct	Importance
1	Perceived social support	0.0159	Body-image concern	0.0093
2	Body-image concern	0.0123	Perceived social support	0.0075
3	Smartphone dependency	0.0123	Smartphone dependency	0.0074
4	Breakfast skipping	0.0098	Waist circumference	0.0035
5	Social jetlag	0.0068	Weighted average sedentary time	0.0034
6	Sleep duration	0.0052	Social jetlag	0.0030
7	Waist circumference	0.0044	Recreational screen time	0.0025
8	Weighted average sedentary time	0.0043	Monthly living allowance	0.0024
9	Body mass index	0.0040	Sleep duration	0.0021
10	Evening chronotype	0.0038	Body mass index	0.0021

Permutation importance was based on AUROC and summarized variable-level decreases. This table reports construct-level sums of related variable-level values; the external joint group-permutation analysis is reported separately in Supplementary Table S21.

**Table 5:** External-validation performance of the primary Feature set C random-forest models.

Outcome	*n*	Positive *n* (%)	AUROC (95% CI)	PR-AUC (95% CI)	Brier score (95% CI)
Depression risk	1,468	148 (10.1%)	0.813 (0.776–0.848)	0.402 (0.325–0.491)	0.090 (0.083–0.098)
Anxiety risk	1,468	79 (5.4%)	0.809 (0.765–0.851)	0.205 (0.144–0.300)	0.063 (0.056–0.070)

PR-AUC was calculated as average precision. Ordinary participant-level bootstrap was the primary external uncertainty analysis; stratified participant-level bootstrap was a sensitivity analysis. Calibration intercept and slope were estimated using logistic calibration models based on the logit of predicted probabilities.

Directionality summaries suggested that higher-ranked constructs showed directionally consistent differences in predicted risk probabilities, including patterns involving smartphone dependency, body-image concern, social jetlag, breakfast skipping, and lower social support. Lower social-support scores and shorter weighted sleep duration also appeared in higher-risk prediction patterns. Waist circumference and monthly allowance required cautious interpretation because their directions were less straightforward or potentially non-linear. Top constructs are shown in Table 4 and Figure 4.

**Figure 4:**
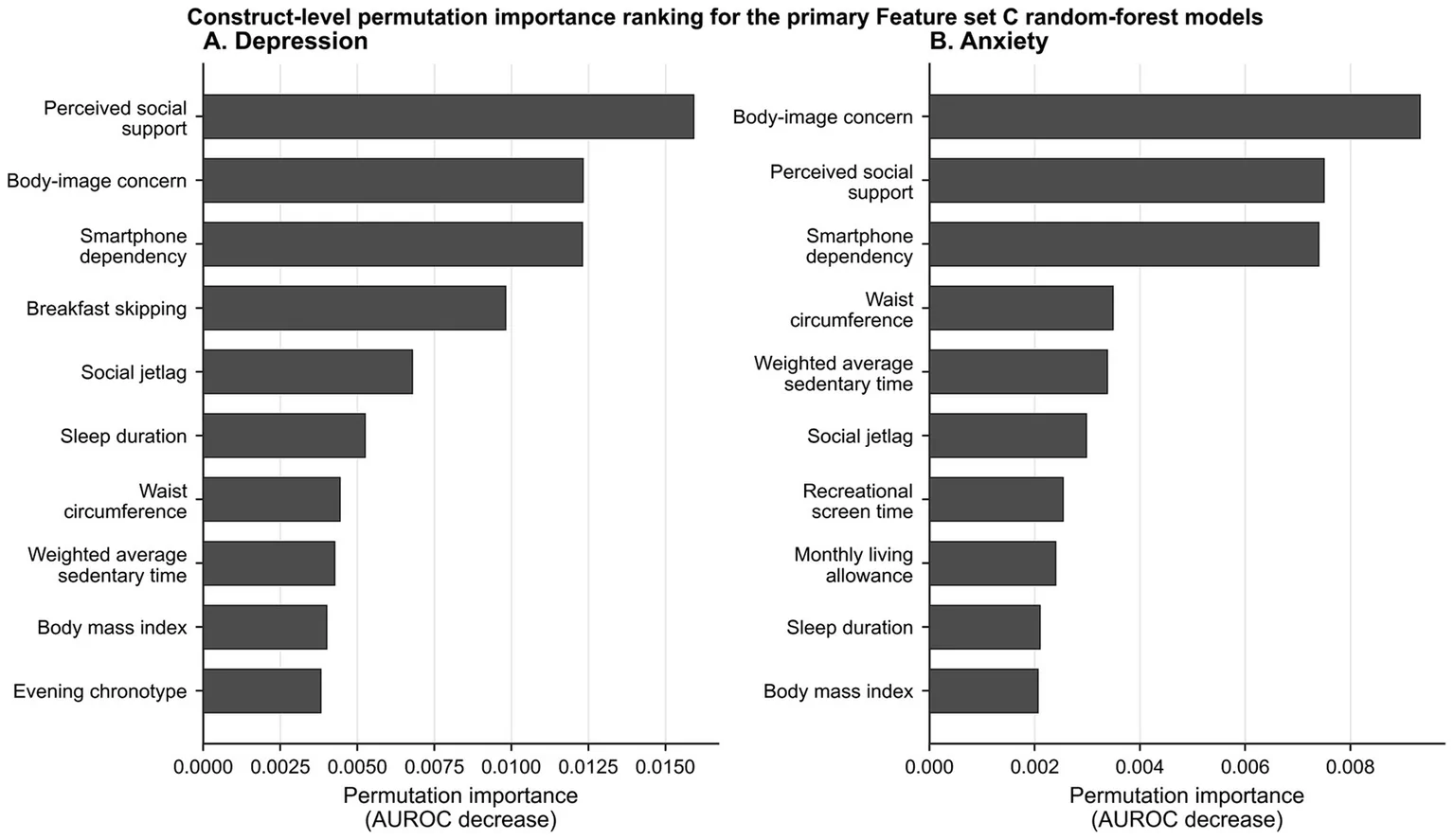
Construct-level permutation-importance patterns for the primary Feature set C random-forest models: **(A)** depression risk and **(B)** anxiety risk. Related variables were summarized by summing their variable-level permutation-importance values; the external joint group-permutation analysis is reported separately in Supplementary Table S21. Values represent decreases in AUROC and are descriptive model-level contributions rather than causal or clinical effects.

### Sensitivity analyses

The legacy internal sensitivity-analysis estimates in Supplementary Table S7 were not retained as formal results after correction of the preprocessing workflow. In the Feature set C-clean analysis, which retained short sleep duration and high social jetlag but removed other dichotomized indicators duplicated by continuous scores, external validation remained similar for depression risk (AUROC = 0.820, PR-AUC = 0.407, Brier score = 0.090) and anxiety risk (AUROC = 0.807, PR-AUC = 0.214, Brier score = 0.062), although default-threshold recall was lower (Supplementary Table S11, Part B). Logistic-regression robustness models showed lower discrimination than random forests, particularly for depression risk. In the Feature set D external-validation sensitivity analysis, AUROC was 0.845 (95% CI, 0.812–0.874), PR-AUC was 0.465 (0.382–0.545), and Brier score was 0.080 (0.073–0.087) for depression risk; corresponding values for anxiety risk were 0.854 (0.810–0.891), 0.278 (0.196–0.389), and 0.053 (0.047–0.060), respectively. These models showed higher AUROC and PR-AUC but greater complexity, so Feature set D remained a sensitivity model (Supplementary Table S25). Anxiety threshold and predictor-importance estimates were treated as exploratory because fewer than 100 positive cases were available. Reciprocal validation showed broadly consistent discrimination for depression risk (AUROC = 0.760, PR-AUC = 0.299, Brier score = 0.076) and anxiety risk (AUROC = 0.823, PR-AUC = 0.272, Brier score = 0.050) (Supplementary Table S12). Depression-risk and anxiety-risk outcomes were strongly associated but not identical (Supplementary Table S13).

### External validation

The external-validation dataset included 1,468 students after duplicate-record exclusion and completeness screening. Depression-risk status was positive in 148 students (10.1%), and anxiety-risk status was positive in 79 students (5.4%). In external validation, the depression-risk model achieved AUROC = 0.813 (95% CI, 0.776–0.848), PR-AUC = 0.402 (95% CI, 0.325–0.491), and Brier score = 0.090 (95% CI, 0.083–0.098) (Figure 5). The anxiety-risk model achieved AUROC = 0.809 (95% CI, 0.765–0.851), PR-AUC = 0.205 (95% CI, 0.144–0.300), and the Brier score = 0.063 (95% CI, 0.056–0.070). Ordinary participant-level bootstrap was the primary uncertainty analysis, with stratified bootstrap performed as a sensitivity analysis. Depression calibration was summarized by an intercept of −0.668 and a slope of 1.401, with a Brier score close to the prevalence-only baseline (0.091). Anxiety showed meaningful external miscalibration: the Brier score was 0.063 versus a prevalence-only baseline of 0.051, with an intercept of −1.227 and a slope of 1.102 (Figure 6). The models therefore supported external risk discrimination and ranking, not validated individual absolute-risk probabilities or clinical decision thresholds. At the default threshold, recall/specificity were 0.318/0.961 for depression risk and 0.241/0.970 for anxiety risk. At the fixed internal Youden thresholds, they were 0.791/0.689 and 0.608/0.828, respectively.

**Figure 5:**
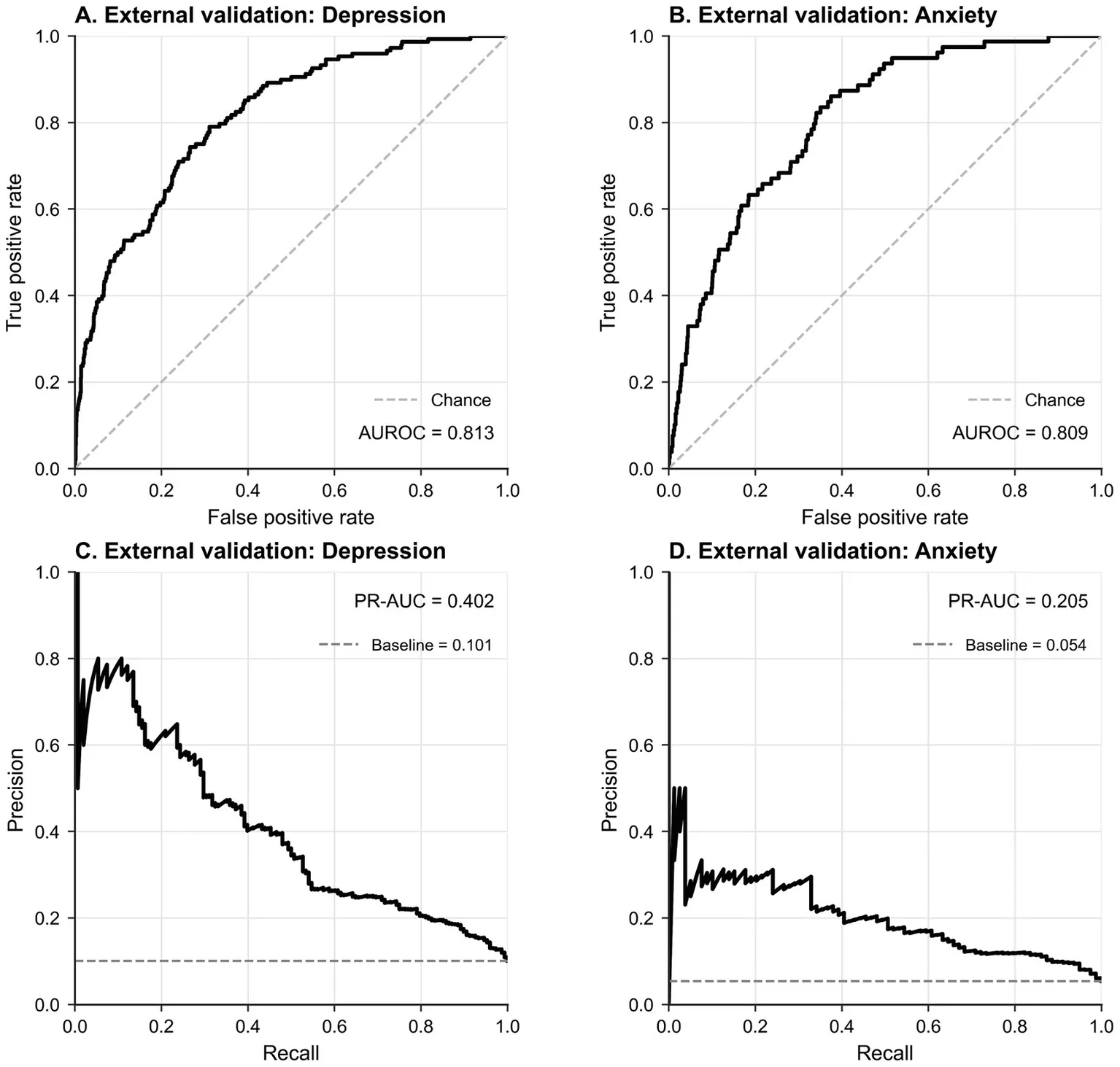
External-validation ROC and precision-recall curves for the primary Feature set C random-forest models. (A,B) ROC curves for depression risk and anxiety risk, respectively; (C,D) precision-recall curves for depression risk and anxiety risk, respectively. Dashed diagonal lines indicate chance discrimination, and dashed horizontal lines in the precision-recall panels indicate outcome prevalence. Exact point estimates and 95% confidence intervals are reported in Table 5 and Supplementary Table S14a.

**Figure 6:**
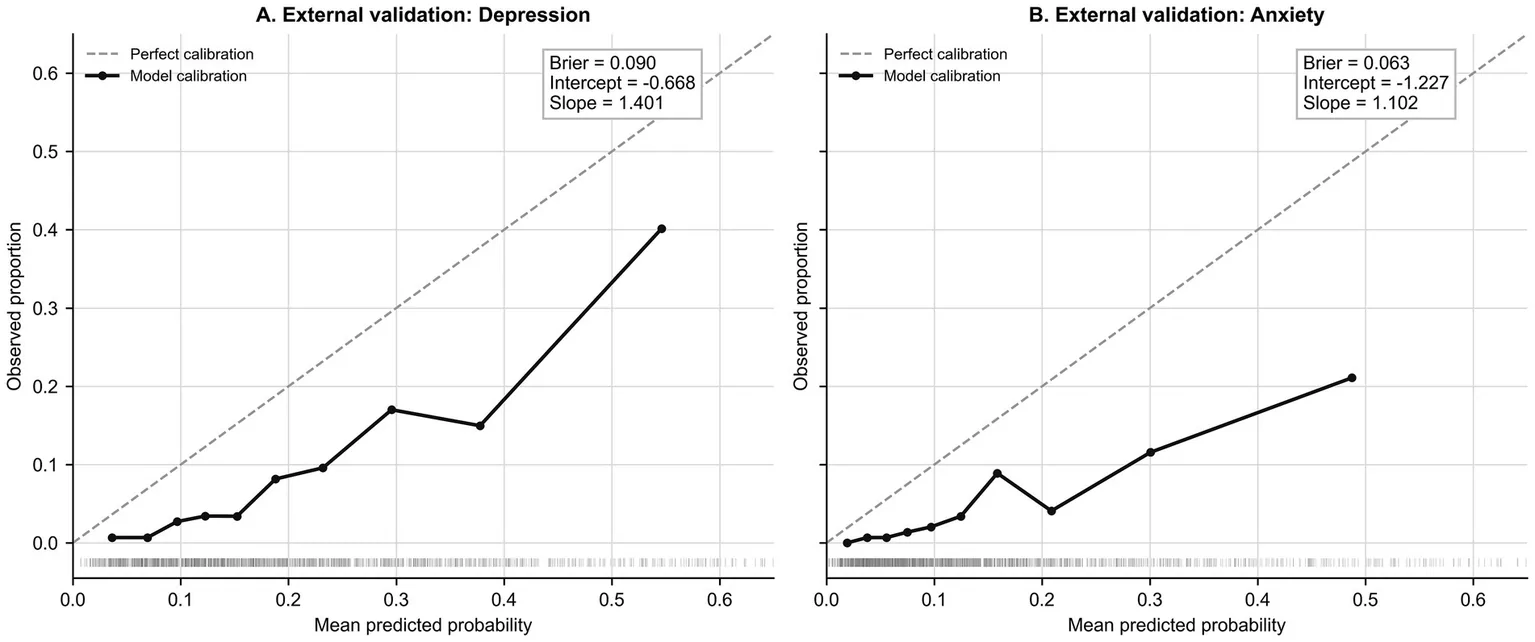
External-validation calibration curves for the uncalibrated primary Feature set C random-forest models: **(A)** depression risk and **(B)** anxiety risk. Calibration curves based on ten quantile bins for the locked external predictions from the primary Feature set C random-forest models are shown on the same probability scale. Dashed diagonal lines indicate perfect calibration, and the rug along the x-axis shows the empirical distribution of individual predicted probabilities. Calibration point estimates and 95% confidence intervals are reported in Supplementary Table S14a.

### Development-only optimization reassessment

The original development-only nested optimization outputs were reviewed after correction of the preprocessing workflow. Because the earlier pipeline materialized percentile flags before cross-validation, those nested performance estimates are not retained as formal results. The corrected Feature set C internal-validation results are reported in Supplementary Table S20, and the prespecified Feature set C model remains the primary benchmark.

## Discussion

### Principal findings

This study developed and internally validated a behavioral and psychosocial risk-stratification framework with construct-level model explanation in a single-university development sample and was externally evaluated using the locked primary model in an independent university sample. Corrected internal estimates and the original locked external point estimates are reported separately and transparently.

External validation supported the discrimination and risk-ranking capability of the primary Feature set C models in an independent university sample. The ordinary-bootstrap results also showed that discrimination and probability calibration provide different information: the depression Brier score was close to the prevalence-only baseline, whereas the anxiety Brier score exceeded it. The framework should therefore be used as a research-oriented risk-stratification approach rather than as a source of validated individual absolute-risk probabilities or clinical decision thresholds.

### Interpretation of model performance

In low-prevalence settings, a model can show high specificity and accuracy while missing many positive cases; precision–recall metrics therefore provide additional information beyond receiver operating characteristic curve (ROC) analysis (Saito and Rehmsmeier, 2015; Davis and Goadrich, 2006).

### Calibration and practical implications

Calibration and discrimination should be interpreted jointly when assessing externally validated risk-stratification frameworks. Differences between university samples may affect absolute-risk estimates, so local recalibration and threshold assessment remain appropriate before broader implementation.

The archived development-only optimization pathway is not interpreted as a formal performance comparison because its legacy percentile preprocessing preceded cross-validation. The prespecified Feature set C model therefore remains the primary benchmark; feature selection, tuning, calibration, and decision analyses are interpreted in light of the corrected internal-validation analysis.

Accordingly, at the current stage, the models are better viewed as risk-stratification tools for research and hypothesis generation rather than diagnostic tools, automatic risk-classification systems, or stand-alone implementation-ready decision-support systems. Threshold interpretation should remain context-specific: the default 0.5 threshold emphasizes specificity, whereas the development-derived Youden thresholds trade specificity for recall. Prevalence-adjusted or utility-based cutoffs may be preferable in practice, depending on the relative costs of false-negative and false-positive classifications and on referral capacity. Local Platt or isotonic recalibration should be assessed using an independent local sample before broader implementation.

### Predictor patterns

The construct pattern was consistent with a multidimensional view of student mental health. Previous machine-learning studies using behavioral and health-related indicators have similarly suggested that heterogeneous information sources may improve identification of students with elevated psychological risk (Chowdhury et al., 2024; Rahman et al., 2023). Recent studies have further emphasized that integrating multidomain behavioral, psychosocial, and digital indicators may provide complementary information for identifying students at elevated mental health risk (Zhang et al., 2024; Shvetcov et al., 2024). Methodological reviews also emphasize transparent reporting and appropriate validation design in AI-based mental health research (Tornero-Costa et al., 2023). External validation and calibration assessment remain essential before broader implementation (Collins et al., 2024; Moons et al., 2025). Feature set C-clean produced similar patterns after removing duplicated indicators; Feature set C remained primary because it retained gradient and threshold-type information.

Perceived social support may represent an interpersonal resource linked to lower depression, anxiety, and stress symptoms among university students (Guo et al., 2021). Problematic smartphone use may intersect with mental health through sleep quality and peer relationships; evidence in Chinese medical students supports sleep quality as a potential pathway between mobile phone addiction and depression (Feng et al., 2022). Passive-sensing research has also used digital behavioral indicators to predict stress among university students (Shvetcov et al., 2024). Body image has been examined alongside depression and anxiety in Chinese college students (Han et al., 2023), while sleep duration was interpreted using established guidance on sleep duration (Hirshkowitz et al., 2015).

Because several dichotomized predictors were derived from continuous questionnaire-derived scores, feature-importance results should be interpreted as construct-level signals rather than independent effects of each individual variable.

The construct-level importance values in Figure 4 should be interpreted as descriptive contributions to model discrimination rather than as effect sizes or evidence of independent clinical effects. Related variables share information, and the variable-level summaries in Figure 4 are complemented by the joint external group-permutation analysis in Supplementary Table S21. Lower-ranked constructs with small importance values may be unstable, so interpretation focuses on broader construct patterns rather than small differences among lower-ranked variables.

The overlap in construct-level model explanation between the depression-risk and anxiety-risk models is consistent with the close psychological relationship between depressive and anxious symptoms. Shared constructs, including perceived social support, body-image concern, smartphone dependency, social jetlag, and sleep duration, may reflect common behavioral and psychosocial contexts. The two outcomes were modeled separately because their ranking, calibration, and event precision differed; anxiety findings remain preliminary because 79 external positive cases produced greater uncertainty, particularly for PR-AUC, calibration, and threshold metrics.

### Strengths and limitations

The study strengths included sample-flow documentation, duplicate review, exclusion of outcome-leakage variables, prespecified feature sets, repeated internal cross-validation, evaluation of PR-AUC and calibration, model-level explanation, and external validation. The limitations included the cross-sectional design, questionnaire-derived predictors, fewer than 100 anxiety-risk cases, restriction to Chinese university settings, possible common-method bias from self-reported predictors and outcomes collected in the same survey, and outcome-specific missingness. Participants with complete outcome data differed in age and perceived social support from those excluded, so selection bias related to outcome availability cannot be excluded. Differences in age and several behavioral distributions between the two cohorts indicate case-mix shift, which may partly contribute to the observed external calibration drift. Sleep-related predictors had approximately 27.5% missingness in the development cohort. The primary imputation strategy implicitly relied on missingness being adequately handled by observed information; however, students with more irregular lifestyles or greater psychological distress may have been more likely to omit sleep-related items, and a missing-not-at-random mechanism cannot be excluded. The Shanghai external cohort contained no sports-major students: sport = 2 for all retained records denoted non-sports majors, and sports_major was therefore set to 0 for all records. The sports_major predictor was invariant in this cohort, so its transportability across different sports-major compositions could not be assessed separately. Development-sample cutoffs and fixed thresholds may require local reassessment, and local Platt or isotonic recalibration may be needed before implementation. A prediction-only all-0/all-1 mapping sensitivity using the locked pipelines is reported in Supplementary Table S24; the current mapping is based on the confirmed sport = 2 code meaning, and the all-1 scenario is a counterfactual sensitivity analysis. In this prediction-only sensitivity, discrimination was relatively insensitive to the alternative coding, whereas probability calibration showed somewhat greater sensitivity (Supplementary Table S24). Feature set D external-validation sensitivity results using the confirmed sports_major = 0 mapping are reported separately in Supplementary Table S25. Exact invitation denominators, response rates, and a complete reconstruction of the original sampling frame or stratification procedure were unavailable from the retained study records.

## Conclusion

In corrected internal validation, emerging behavioral and psychosocial indicators improved risk stratification beyond demographic and traditional health-behavior variables. External validation showed that the primary Feature set C models retained discrimination and risk-ranking capability, but probability calibration and practical thresholds require further evaluation. The results support a model-level explanatory framework for prevention-oriented research, not a clinical or screening-ready decision system. Anxiety-risk estimates should be interpreted as preliminary because the number of positive cases was limited.

## Data Availability

The participant-level datasets are not publicly available because they contain sensitive health information and are subject to ethical and institutional restrictions. Requests for access to de-identified data should be directed to the corresponding author, Guopeng Hu (hugp@hqu.edu.cn). Access may be granted on reasonable request, subject to the relevant ethical and institutional approvals. The analysis code and locked model objects can be made available from the corresponding author on reasonable request, subject to institutional, ethical, and data-use restrictions.
