# Supplementary material for: Behavioral and psychosocial indicators for depression and anxiety risk stratification among college students: development and external validation of a machine-learning-assisted framework
Source: Front Psychol. 2026 Oct 27;17:1972318. doi: 10.3389/fpsyg.2026.1972318 (PMC13748373; doi:10.3389/fpsyg.2026.1972318)
Supplement: Supplementary file 1 [file Supplementary_file_1.docx]

**Supplementary Materials**

Behavioral and Psychosocial Indicators for Depression and Anxiety Risk Stratification Among College Students: Development and External Validation of a Machine-Learning-Assisted Framework

Authors: Kailin Mao^1^, Yuqi He^1^, Xiaojian Yin^2^, Lihan Lin^3^, Yikun Zheng^1^, Guopeng Hu^1*^

1 School of Physical Education, Huaqiao University, Quanzhou, China

2 Shanghai Institute of Technology, Shanghai, China

3 Provincial University Key Laboratory of Sport and Health Science, School of Physical Education and Sport Science, Fujian Normal University, Fuzhou, China

*Correspondence: Guopeng Hu, School of Physical Education, Huaqiao University, Quanzhou, China; hugp@hqu.edu.cn

**Supplementary Table S1. External-validation duplicate-record handling summary**

| **Item** | **Definition / criterion** | **n** | **Notes** |
| --- | --- | --- | --- |
| Duplicate identification criteria | Student identifier, name, sex, and birth date when available | Not applicable | Complete records were retained preferentially; remaining ties were resolved by data completeness and later record/submission time. |
| Suspected duplicate groups before deduplication | Groups meeting duplicate-review criteria | 336 | Based on the external-validation cleaning report. |
| Suspected duplicate records before deduplication | Records involved in suspected duplicate groups | 789 | Based on the external-validation cleaning report. |
| Duplicate records excluded | Records removed by the duplicate-retention rule | 453 | External sample flow: 1,983 raw records to 1,530 after duplicate-record exclusion. The retained records did not preserve an item-level completeness threshold for the 62 questionnaires; these records were identified only because they were not marked complete in the questionnaire system. |
| Records retained after duplicate exclusion | External records retained after applying the duplicate-retention rule | 1,530 | Completeness screening was applied after duplicate-record exclusion. |
| Complete external-validation dataset | Records retained after completeness screening | 1,468 | Used for both depression-risk and anxiety-risk external validation analyses. |

Note. Duplicate records were handled according to the prespecified retention rule.

**Supplementary Table S2a. Operational definitions, coding, and threshold basis for key predictors**

**Part 1**

| **Variable** | **Operational definition / source item** | **Coding or threshold basis** |
| --- | --- | --- |
| Depression-risk outcome (depression_risk) | PHQ-9 total score. | 1 = PHQ-9 ≥ 10; 0 = PHQ-9 < 10. Used as outcome only, not as predictor. |
| Anxiety-risk outcome (anxiety_risk) | GAD-7 total score. | 1 = GAD-7 ≥ 10; 0 = GAD-7 < 10. Used as outcome only, not as predictor. |
| Physical inactivity (physical_inactivity) | Physical-activity summary derived from reported vigorous, moderate, and walking activity. | 1 = total physical activity <600 MET-min/week, or not meeting the WHO-equivalent recommendation of at least 150 min/week moderate activity, 75 min/week vigorous activity, or an equivalent combination; 0 = otherwise. |
| Weighted average sedentary time (weighted_sedentary_hours) | Weighted average of weekday and weekend sedentary time: (weekday sedentary time × 5 + weekend sedentary time × 2) / 7. | Continuous variable in hours/day. |
| High sedentary time (high_sedentary) | Dichotomized sedentary-time indicator derived from Weighted average sedentary time (weighted_sedentary_hours). | 1 = weighted sedentary time ≥7 h/day; 0 = <7 h/day. |
| Breakfast skipping (breakfast_skipping) | Breakfast frequency on school days. | 1 = breakfast consumed on fewer than 5 school days/week; 0 = breakfast consumed on all 5 school days/week. |
| Unhealthy dietary-risk index (unhealthy_diet_score) | Composite score from six dietary-behavior items: no vegetables, no fruit, sweet food, fried food, fast food, and daily sugar-sweetened beverage intake. The retained codebook does not provide more granular intake-adequacy thresholds for the no-vegetables and no-fruit response categories. | Sum of six binary components; score calculated when at least four components were non-missing. |
| High unhealthy dietary risk (high_unhealthy_diet) | Dichotomized dietary-risk indicator derived from Unhealthy dietary-risk index (unhealthy_diet_score). | 1 = unhealthy dietary-risk index ≥3; 0 = <3. The score used six components and was calculated when at least four components were non-missing. |
| Current smoking (current_smoking) | Current cigarette-smoking status. | 1 = d4 ≥2 (smoked on at least one day in the past month), unless smoking-habit code d3 indicates no smoking; 0 = otherwise. |
| Current alcohol drinking (current_drinking) | Current alcohol-drinking status. | 1 = d1 ≥2 (drank alcohol on at least one day in the past month); 0 = otherwise. |
| Binge drinking (binge_drinking) | Binge-drinking item. | 1 = d2 ≥2 (binge drinking on at least one day in the past month); set to 0 when d1 = 1. |
| Weighted average sleep duration (weighted_sleep_duration) | Weighted average of weekday and weekend sleep duration: (weekday sleep duration × 5 + weekend sleep duration × 2) / 7. | Continuous variable in hours/night. |
| Short sleep duration (short_sleep) | Dichotomized sleep-duration indicator derived from Weighted average sleep duration (weighted_sleep_duration). | 1 = weighted sleep duration <7 h/night; 0 = ≥7 h/night. |
| Recreational screen time (recreational_screen_time_hours) | Self-reported recreational screen time outside study/work use. | Continuous variable in hours/day. |
| Late dinner (late_dinner) | Late dinner behavior reported in the dietary-rhythm section of the questionnaire. | 1 = dinner after 20:00 on ≥3 days/week; 0 = otherwise. |
| Daily sugar-sweetened beverage intake (daily_ssb) | Sugar-sweetened beverage intake frequency. | 1 = at least 1 sugar-sweetened beverage serving/day; 0 = <1 serving/day. |

**Part 2**

| **Variable** | **Operational definition / source item** | **Coding or threshold basis** |
| --- | --- | --- |
| High recreational screen time (high_recreational_screen) | Dichotomized recreational screen-time indicator derived from Recreational screen time (recreational_screen_time_hours). | 1 = recreational screen time ≥4 h/day; 0 = <4 h/day. |
| Smartphone dependency score (smartphone_dependency_score) | Composite smartphone-dependency score derived from questionnaire items. | Continuous score; higher values indicate greater smartphone dependency. |
| High smartphone dependency (high_smartphone_dependency) | Dichotomized smartphone-dependency indicator derived from Smartphone dependency score (smartphone_dependency_score). | 1 = smartphone dependency score ≥29.0; 0 = <29.0, using the fixed development-sample cutoff. |
| Night-light exposure index (night_light_exposure_score) | Composite night-light exposure score derived from bedroom-light and bedtime/nighttime phone-use items. | Continuous score; higher values indicate greater night-light exposure. |
| High night-light exposure (high_night_light_exposure) | Dichotomized night-light exposure indicator derived from Night-light exposure index (night_light_exposure_score). | 1 = night-light exposure index ≥4; 0 = <4. |
| Social jetlag (social_jetlag) | Absolute difference between weekday and weekend sleep midpoint. | Continuous variable in hours. |
| High social jetlag (high_social_jetlag) | Dichotomized social-jetlag indicator derived from Social jetlag (social_jetlag). | 1 = social jetlag >2 h; 0 = ≤2 h. |
| Evening chronotype (evening_type) | Chronotype or late-schedule category derived from sleep-rhythm questionnaire items. | 1 = i5 ≤2 (evening or definite-evening response); 0 = i5 >2; missing if i5 is missing. |
| Body-image concern score (body_image_concern_score) | Composite body-image concern score derived from body-image questionnaire items. | Continuous score; higher values indicate greater body-image concern. |
| High body-image concern (high_body_image_concern) | Dichotomized body-image concern indicator derived from Body-image concern score (body_image_concern_score). | 1 = body-image concern score ≥25.0; 0 = <25.0, using the fixed development-sample cutoff. |
| Perceived social support score (social_support_score) | Composite social-support score derived from questionnaire items. | Continuous score; higher values indicate greater perceived social support. |
| Low perceived social support (low_social_support) | Dichotomized social-support indicator derived from Perceived social support score (social_support_score). | 1 = perceived social support score ≤51.0; 0 = >51.0, using the fixed development-sample cutoff. |
| Number of friends (number_of_friends) | Self-reported number of close friends or friend-category response. | Ordinal/categorical variable coded according to original questionnaire response options; higher categories indicate more close friends. |
| Student-organization participation (student_organization_participation) | Participation in student organizations or campus activities. | 1 = yes; 0 = no. |

Note. Coding definitions correspond to the prespecified feature-engineering procedures used in the analysis.

**Supplementary Table S2b. Predictor feature-set membership and missingness**

**Feature set A**

| **Feature set** | **Order** | **Variable** | **Missing %** | **Role** |
| --- | --- | --- | --- | --- |
| A | 1 | Age (age_years) | 4.18 | main_model |
| A | 2 | Sex (sex) | 3.71 | main_model |
| A | 3 | Academic year (grade) | 0.39 | main_model |
| A | 4 | Sports-major status (sports_major) | 0.39 | main_model |
| A | 5 | Body mass index (bmi) | 5.76 | main_model |
| A | 6 | Waist circumference (waist_cm) | 6.31 | main_model |
| A | 7 | Residence (residence) | 6.31 | main_model |
| A | 8 | Monthly living allowance (monthly_allowance) | 6.63 | main_model |
| A | 9 | Higher parental education (parental_education_high) | 6.63 | main_model |
| A | 10 | Only-child status (only_child) | 6.47 | main_model |

**Feature set B**

| **Feature set** | **Order** | **Variable** | **Missing %** | **Role** |
| --- | --- | --- | --- | --- |
| B | 1 | Age (age_years) | 4.18 | main_model |
| B | 2 | Sex (sex) | 3.71 | main_model |
| B | 3 | Academic year (grade) | 0.39 | main_model |
| B | 4 | Sports-major status (sports_major) | 0.39 | main_model |
| B | 5 | Body mass index (bmi) | 5.76 | main_model |
| B | 6 | Waist circumference (waist_cm) | 6.31 | main_model |
| B | 7 | Residence (residence) | 6.31 | main_model |
| B | 8 | Monthly living allowance (monthly_allowance) | 6.63 | main_model |
| B | 9 | Higher parental education (parental_education_high) | 6.63 | main_model |
| B | 10 | Only-child status (only_child) | 6.47 | main_model |
| B | 11 | Physical inactivity (physical_inactivity) | 9.31 | main_model |
| B | 12 | Weighted average sedentary time (weighted_sedentary_hours) | 9.39 | main_model |
| B | 13 | High sedentary time (high_sedentary) | 9.39 | main_model |
| B | 14 | Breakfast skipping (breakfast_skipping) | 11.29 | main_model |
| B | 15 | Late dinner (late_dinner) | 11.29 | main_model |
| B | 16 | Daily sugar-sweetened beverage intake (daily_ssb) | 9.00 | main_model |
| B | 17 | Unhealthy dietary-risk index (unhealthy_diet_score) | 8.68 | main_model |
| B | 18 | High unhealthy dietary risk (high_unhealthy_diet) | 8.68 | main_model |
| B | 19 | Current smoking (current_smoking) | 8.13 | main_model |
| B | 20 | Current alcohol drinking (current_drinking) | 8.13 | main_model |
| B | 21 | Binge drinking (binge_drinking) | 8.13 | main_model |
| B | 22 | Short sleep duration (short_sleep) | 27.47 | main_model |
| B | 23 | Weighted average sleep duration (weighted_sleep_duration) | 27.47 | main_model |

**Feature set C (part 1)**

| **Feature set** | **Order** | **Variable** | **Missing %** | **Role** |
| --- | --- | --- | --- | --- |
| C | 1 | Age (age_years) | 4.18 | main_model |
| C | 2 | Sex (sex) | 3.71 | main_model |
| C | 3 | Academic year (grade) | 0.39 | main_model |
| C | 4 | Sports-major status (sports_major) | 0.39 | main_model |
| C | 5 | Body mass index (bmi) | 5.76 | main_model |
| C | 6 | Waist circumference (waist_cm) | 6.31 | main_model |
| C | 7 | Residence (residence) | 6.31 | main_model |
| C | 8 | Monthly living allowance (monthly_allowance) | 6.63 | main_model |
| C | 9 | Higher parental education (parental_education_high) | 6.63 | main_model |
| C | 10 | Only-child status (only_child) | 6.47 | main_model |
| C | 11 | Physical inactivity (physical_inactivity) | 9.31 | main_model |
| C | 12 | Weighted average sedentary time (weighted_sedentary_hours) | 9.39 | main_model |
| C | 13 | High sedentary time (high_sedentary) | 9.39 | main_model |
| C | 14 | Breakfast skipping (breakfast_skipping) | 11.29 | main_model |
| C | 15 | Late dinner (late_dinner) | 11.29 | main_model |
| C | 16 | Daily sugar-sweetened beverage intake (daily_ssb) | 9.00 | main_model |
| C | 17 | Unhealthy dietary-risk index (unhealthy_diet_score) | 8.68 | main_model |
| C | 18 | High unhealthy dietary risk (high_unhealthy_diet) | 8.68 | main_model |

**Feature set C (part 2)**

| **Feature set** | **Order** | **Variable** | **Missing %** | **Role** |
| --- | --- | --- | --- | --- |
| C | 19 | Current smoking (current_smoking) | 8.13 | main_model |
| C | 20 | Current alcohol drinking (current_drinking) | 8.13 | main_model |
| C | 21 | Binge drinking (binge_drinking) | 8.13 | main_model |
| C | 22 | Short sleep duration (short_sleep) | 27.47 | main_model |
| C | 23 | Weighted average sleep duration (weighted_sleep_duration) | 27.47 | main_model |
| C | 24 | Recreational screen time (recreational_screen_time_hours) | 8.21 | main_model |
| C | 25 | High recreational screen time (high_recreational_screen) | 8.21 | main_model |
| C | 26 | Smartphone dependency score (smartphone_dependency_score) | 7.97 | main_model |
| C | 27 | High smartphone dependency (high_smartphone_dependency) | 7.97 | main_model |
| C | 28 | Night-light exposure index (night_light_exposure_score) | 7.42 | main_model |
| C | 29 | High night-light exposure (high_night_light_exposure) | 7.42 | main_model |
| C | 30 | Social jetlag (social_jetlag) | 27.47 | main_model |
| C | 31 | High social jetlag (high_social_jetlag) | 27.47 | main_model |
| C | 32 | Evening chronotype (evening_type) | 11.21 | main_model |
| C | 33 | Body-image concern score (body_image_concern_score) | 11.37 | main_model |
| C | 34 | High body-image concern (high_body_image_concern) | 11.37 | main_model |
| C | 35 | Perceived social support score (social_support_score) | 11.84 | main_model |
| C | 36 | Low perceived social support (low_social_support) | 11.84 | main_model |

**Feature set C (part 3)**

| **Feature set** | **Order** | **Variable** | **Missing %** | **Role** |
| --- | --- | --- | --- | --- |
| C | 37 | Number of friends (number_of_friends) | 12.00 | main_model |
| C | 38 | Student-organization participation (student_organization_participation) | 12.08 | main_model |

**Feature set D (part 1)**

| **Feature set** | **Order** | **Variable** | **Missing %** | **Role** |
| --- | --- | --- | --- | --- |
| D | 1 | Age (age_years) | 4.18 | sensitivity_only |
| D | 2 | Sex (sex) | 3.71 | sensitivity_only |
| D | 3 | Academic year (grade) | 0.39 | sensitivity_only |
| D | 4 | Sports-major status (sports_major) | 0.39 | sensitivity_only |
| D | 5 | Height (height_cm) | 5.76 | sensitivity_only |
| D | 6 | Weight (weight_kg) | 5.76 | sensitivity_only |
| D | 7 | Body mass index (bmi) | 5.76 | sensitivity_only |
| D | 8 | Waist circumference (waist_cm) | 6.31 | sensitivity_only |
| D | 9 | Residence (residence) | 6.31 | sensitivity_only |
| D | 10 | Monthly living allowance (monthly_allowance) | 6.63 | sensitivity_only |
| D | 11 | Father education (father_education) | 6.63 | sensitivity_only |
| D | 12 | Mother education (mother_education) | 6.63 | sensitivity_only |
| D | 13 | Higher parental education (parental_education_high) | 6.63 | sensitivity_only |
| D | 14 | Only-child status (only_child) | 6.47 | sensitivity_only |
| D | 15 | Vigorous activity days (vigorous_days) | 9.31 | sensitivity_only |
| D | 16 | Vigorous activity minutes (vigorous_minutes) | 9.39 | sensitivity_only |
| D | 17 | Moderate activity days (moderate_days) | 9.39 | sensitivity_only |
| D | 18 | Moderate activity minutes (moderate_minutes) | 9.39 | sensitivity_only |

**Feature set D (part 2)**

| **Feature set** | **Order** | **Variable** | **Missing %** | **Role** |
| --- | --- | --- | --- | --- |
| D | 19 | Walking days (walking_days) | 9.39 | sensitivity_only |
| D | 20 | Walking minutes (walking_minutes) | 9.39 | sensitivity_only |
| D | 21 | Total physical activity (total_MET_min_week) | 9.39 | sensitivity_only |
| D | 22 | Physical inactivity (physical_inactivity) | 9.31 | sensitivity_only |
| D | 23 | Weekday sedentary time (weekday_sedentary_minutes) | 9.39 | sensitivity_only |
| D | 24 | Weekend sedentary time (weekend_sedentary_minutes) | 9.39 | sensitivity_only |
| D | 25 | Weighted average sedentary time (weighted_sedentary_hours) | 9.39 | sensitivity_only |
| D | 26 | High sedentary time (high_sedentary) | 9.39 | sensitivity_only |
| D | 27 | Learning screen time (learning_screen_time_hours) | 7.81 | sensitivity_only |
| D | 28 | Recreational screen time (recreational_screen_time_hours) | 8.21 | sensitivity_only |
| D | 29 | High recreational screen time (high_recreational_screen) | 8.21 | sensitivity_only |
| D | 30 | Smartphone dependency score (smartphone_dependency_score) | 7.97 | sensitivity_only |
| D | 31 | High smartphone dependency (high_smartphone_dependency) | 7.97 | sensitivity_only |
| D | 32 | Sleeping with light exposure (sleep_with_light) | 7.34 | sensitivity_only |
| D | 33 | Bedroom light level (bedroom_light_level) | 7.42 | sensitivity_only |
| D | 34 | Curtain use (curtain_use) | 7.42 | sensitivity_only |
| D | 35 | Bedtime phone use (bedtime_phone_use) | 7.50 | sensitivity_only |
| D | 36 | Nighttime phone use (nighttime_phone_use) | 7.50 | sensitivity_only |

**Feature set D (part 3)**

| **Feature set** | **Order** | **Variable** | **Missing %** | **Role** |
| --- | --- | --- | --- | --- |
| D | 37 | Night-light exposure index (night_light_exposure_score) | 7.42 | sensitivity_only |
| D | 38 | High night-light exposure (high_night_light_exposure) | 7.42 | sensitivity_only |
| D | 39 | Weekday sleep duration (weekday_sleep_duration) | 23.52 | sensitivity_only |
| D | 40 | Weekend sleep duration (weekend_sleep_duration) | 22.18 | sensitivity_only |
| D | 41 | Weighted average sleep duration (weighted_sleep_duration) | 27.47 | sensitivity_only |
| D | 42 | Short sleep duration (short_sleep) | 27.47 | sensitivity_only |
| D | 43 | Weekday sleep midpoint (weekday_sleep_midpoint) | 23.52 | sensitivity_only |
| D | 44 | Weekend sleep midpoint (weekend_sleep_midpoint) | 22.18 | sensitivity_only |
| D | 45 | Social jetlag (social_jetlag) | 27.47 | sensitivity_only |
| D | 46 | High social jetlag (high_social_jetlag) | 27.47 | sensitivity_only |
| D | 47 | Evening chronotype (evening_type) | 11.21 | sensitivity_only |
| D | 48 | Breakfast skipping (breakfast_skipping) | 11.29 | sensitivity_only |
| D | 49 | Late dinner (late_dinner) | 11.29 | sensitivity_only |
| D | 50 | Daily sugar-sweetened beverage intake (daily_ssb) | 9.00 | sensitivity_only |
| D | 51 | Unhealthy dietary-risk index (unhealthy_diet_score) | 8.68 | sensitivity_only |
| D | 52 | High unhealthy dietary risk (high_unhealthy_diet) | 8.68 | sensitivity_only |
| D | 53 | Current smoking (current_smoking) | 8.13 | sensitivity_only |
| D | 54 | Current alcohol drinking (current_drinking) | 8.13 | sensitivity_only |

**Feature set D (part 4)**

| **Feature set** | **Order** | **Variable** | **Missing %** | **Role** |
| --- | --- | --- | --- | --- |
| D | 55 | Binge drinking (binge_drinking) | 8.13 | sensitivity_only |
| D | 56 | Secondhand smoke exposure (secondhand_smoke) | 8.13 | sensitivity_only |
| D | 57 | Body-image concern score (body_image_concern_score) | 11.37 | sensitivity_only |
| D | 58 | High body-image concern (high_body_image_concern) | 11.37 | sensitivity_only |
| D | 59 | Perceived social support score (social_support_score) | 11.84 | sensitivity_only |
| D | 60 | Low perceived social support (low_social_support) | 11.84 | sensitivity_only |
| D | 61 | Number of friends (number_of_friends) | 12.00 | sensitivity_only |
| D | 62 | Student-organization participation (student_organization_participation) | 12.08 | sensitivity_only |
| D | 63 | Recent illness (recent_illness) | 12.39 | sensitivity_only |
| D | 64 | Sickness absence (sickness_absence) | 12.55 | sensitivity_only |
| D | 65 | Chronic disease count (chronic_disease_count) | 12.23 | sensitivity_only |
| D | 66 | Myopia status (myopia_status) | 12.55 | sensitivity_only |
| D | 67 | Neck/shoulder pain score (neck_shoulder_pain_score) | 12.63 | sensitivity_only |
| D | 68 | Low back pain score (low_back_pain_score) | 12.63 | sensitivity_only |
| D | 69 | Snoring (snoring) | 12.79 | sensitivity_only |
| D | 70 | Observed sleep apnea (observed_sleep_apnea) | 12.79 | sensitivity_only |

Note. This table lists feature-set membership and missingness for candidate predictors. Variable coding follows the prespecified feature-engineering procedures used in the analysis scripts.

**Supplementary Table S2c. Measurement characteristics of questionnaire-derived composite scores**

| **Composite score** | **Construct or index** | **Item/component basis** | **Items/components** | **Observed range** | **Higher score indicates** | **Reliability or rationale** | **Source basis** |
| --- | --- | --- | --- | --- | --- | --- | --- |
| Smartphone dependency score (smartphone_dependency_score) | Smartphone dependency (reflective questionnaire-derived score) | Direct sum of observed c5_1-c5_13 item values; score calculated when at least 10 of 13 items were non-missing. Missing items were neither imputed nor prorated at score-construction stage. | 13 items | 13-65 (non-missing n=1166) | Greater smartphone dependency | Cronbach's alpha = 0.918 (complete-case n=1164) | Questionnaire-derived composite score |
| Body-image concern score (body_image_concern_score) | Body-image concern (reflective questionnaire-derived score) | Direct sum of observed k1_1-k1_8 item values; score calculated when at least 6 of 8 items were non-missing. Missing items were neither imputed nor prorated at score-construction stage. | 8 items | 8-48 (non-missing n=1123) | Greater body-image concern | Cronbach's alpha = 0.942 (complete-case n=1123) | Questionnaire-derived composite score |
| Perceived social support score (social_support_score) | Perceived social support (reflective questionnaire-derived score) | Direct sum of observed l1_1-l1_12 item values; score calculated when at least 9 of 12 items were non-missing. Missing items were neither imputed nor prorated at score-construction stage. | 12 items | 12-84 (non-missing n=1117) | Greater perceived social support | Cronbach's alpha = 0.941 (complete-case n=1115) | Questionnaire-derived composite score |
| Unhealthy dietary-risk index (unhealthy_diet_score) | Unhealthy dietary-risk index (formative behavioral risk index) | Direct sum of six observed binary components: no vegetables, no fruit, sweet food, fried food, fast food, and daily sugar-sweetened beverage intake; score calculated when at least four components were non-missing. | 6 components | 0-6 (non-missing n=1157) | More unhealthy dietary risk components | Not calculated; formative behavioral risk index. | Questionnaire-derived formative behavioral risk index |
| Night-light exposure index (night_light_exposure_score) | Night-light exposure index (formative exposure index) | Five binary components: sleep with light, bedroom brightness, poor curtain/light blocking, bedtime phone use, and nighttime phone use. | 5 components | 0-5 (non-missing n=1173) | Greater night-light exposure | Not calculated; formative exposure index. | Questionnaire-derived formative exposure index |

Note. Observed ranges were calculated in the cleaned development analytic dataset. Cronbach alpha was calculated for reflective questionnaire-derived scores with identifiable component items; reliability coefficients were not calculated for formative behavioral risk or exposure indices.

**Supplementary Table S3. Feature-set missingness summary**

| **Feature set** | **Included variables** | **Variables >30% missing** | **Maximum missing %** | **High-missingness variables** |
| --- | --- | --- | --- | --- |
| A | 10 | 0 | 6.63 |  |
| B | 23 | 0 | 27.47 |  |
| C | 38 | 0 | 27.47 |  |
| D | 70 | 0 | 27.47 |  |

**Supplementary Table S4. Algorithm comparison for Feature set C**

| **Outcome** | **Model** | **AUROC (corrected pooled CV)** | **PR-AUC (corrected pooled CV)** | **Brier (corrected pooled CV)** | **Recall** | **Specificity** |
| --- | --- | --- | --- | --- | --- | --- |
| Depression risk | Logistic Regression | 0.745 | 0.252 | 0.179 | 0.588 | 0.743 |
| Depression risk | Random Forest | 0.773 | 0.266 | 0.091 | 0.176 | 0.963 |
| Depression risk | Support Vector Machine | 0.708 | 0.191 | 0.081 | 0.000 | 0.997 |
| Anxiety risk | Logistic Regression | 0.786 | 0.217 | 0.150 | 0.561 | 0.784 |
| Anxiety risk | Random Forest | 0.796 | 0.266 | 0.062 | 0.167 | 0.984 |
| Anxiety risk | Support Vector Machine | 0.748 | 0.216 | 0.052 | 0.000 | 1.000 |

**Supplementary Table S5. Model specifications and hyperparameters**

Model parameters were prespecified. Unless listed below, scikit-learn default settings were used. No resampling methods (SMOTE, oversampling, or undersampling) were applied. Randomized-search hyperparameter optimization was performed only for the separate development-only optimization analysis.

| **Component** | **Implementation** | **Key settings** | **Use in analysis** |
| --- | --- | --- | --- |
| Cross-validation | RepeatedStratifiedKFold | 5 folds, 5 repeats, random_state = 2026; validation folds kept outcome-stratified | Corrected internal-validation estimates only; external validation used the locked development pipelines separately. |
| Preprocessing pipeline | ColumnTransformer with SimpleImputer, StandardScaler, and OneHotEncoder | Numeric variables: median imputation and standardization; categorical variables: most-frequent imputation and one-hot encoding with unseen categories ignored | Fitted inside each training fold only |
| Logistic regression | sklearn.linear_model.LogisticRegression | L2 penalty, C = 1e6, solver = lbfgs, max_iter = 3000, class_weight = balanced | Algorithm comparison and robustness analysis |
| Random Forest | sklearn.ensemble.RandomForestClassifier | n_estimators = 300, criterion = gini, max_depth = None, max_features = sqrt, min_samples_leaf = 5, random_state = 2026, class_weight = balanced, n_jobs = 1 | Primary algorithm for Feature set C and sensitivity analyses; consistent with the saved model objects and original modeling scripts |
| Support vector machine | sklearn.svm.SVC | kernel = rbf, C = 1.0, gamma = scale, probability = True, class_weight = None, random_state = 2026 | Algorithm comparison for Feature set C |
| Threshold analysis | Predicted probabilities from validation folds | Default threshold = 0.5; Youden threshold chosen from validation predictions for descriptive threshold trade-off analysis | Threshold-dependent metrics |
| Primary internal calibration assessment | Uncalibrated corrected Feature set C participant-level OOF probabilities | Arithmetic mean of each participant's five held-out probabilities before evaluation; Brier score, calibration intercept and slope, and ten quantile-binned calibration curves. | Formal corrected internal calibration. Pre-correction sigmoid/isotonic comparisons are not retained as formal corrected results. |
| Variable-level and construct-level descriptive model explanation | Permutation importance | Permutation importance based on AUROC; 10 repeats; directionality assessed descriptively from prediction patterns | Top-predictor ranking and interpretation |

Note. Primary model implementation.

**Supplementary Table S6. External validation sleep-derived variable availability after preprocessing correction**

| **External sleep-derived variable** | **Non-missing n / N** | **Notes** |
| --- | --- | --- |
| Weighted average sleep duration (weighted_sleep_duration) | 1462 / 1468 | Derived after converting seconds-since-midnight sleep-time variables to clock-time values. |
| Short sleep duration (short_sleep) | 1462 / 1468 | Derived from Weighted average sleep duration (weighted_sleep_duration) according to the development-sample coding rule. |
| Social jetlag (social_jetlag) | 1462 / 1468 | Derived after converting weekday and weekend sleep midpoint variables. |
| High social jetlag (high_social_jetlag) | 1462 / 1468 | Derived from Social jetlag (social_jetlag) according to the development-sample coding rule. |

Note. The external validation dataset encoded sleep-time variables as seconds since midnight. These values were converted to clock time before deriving sleep-duration and social-jetlag indicators. Sleep-derived variables were generated using the same preprocessing rules applied in the development dataset.

**Supplementary Table S7. Legacy sensitivity analyses**

Depression risk

| **Outcome** | **Scenario** | **Model** | **n** | **Positive n** | **AUROC** | **PR-AUC** | **ΔAUROC** | **ΔPR-AUC** |
| --- | --- | --- | --- | --- | --- | --- | --- | --- |
| Depression risk | Legacy sensitivity artifacts | Not retained as formal estimates | 1096 | 102 | Not retained | Not retained | Not retained | Not retained |

Anxiety risk

| **Outcome** | **Scenario** | **Model** | **n** | **Positive n** | **AUROC** | **PR-AUC** | **ΔAUROC** | **ΔPR-AUC** |
| --- | --- | --- | --- | --- | --- | --- | --- | --- |
| Anxiety risk | Legacy sensitivity artifacts | Not retained as formal estimates | 1095 | 66 | Not retained | Not retained | Not retained | Not retained |

Note. Legacy internal sensitivity-analysis estimates were not retained as formal results after correction of the preprocessing workflow.

**Supplementary Table S8. Participant characteristics by anxiety-risk status**

Continuous characteristics

| **Characteristic** | **Overall** | **Anxiety-risk negative** | **Anxiety-risk positive** | **P value** |
| --- | --- | --- | --- | --- |
| N | 1095 | 1029 | 66 |  |
| Age, years, mean ± SD | 19.17 ± 1.11 | 19.14 ± 1.08 | 19.65 ± 1.46 | 0.003 |
| BMI, kg/m², mean ± SD | 21.98 ± 4.87 | 22.00 ± 4.91 | 21.69 ± 4.27 | 0.731 |
| Waist circumference, cm, mean ± SD | 76.80 ± 11.25 | 76.94 ± 11.15 | 74.63 ± 12.55 | 0.062 |
| Weighted sedentary hours, mean ± SD | 5.82 ± 3.27 | 5.81 ± 3.29 | 6.01 ± 2.85 | 0.278 |
| Recreational screen time, hours, mean ± SD | 4.09 ± 3.20 | 4.03 ± 3.14 | 4.89 ± 3.97 | 0.107 |
| Smartphone dependency score, mean ± SD | 24.51 ± 8.88 | 23.99 ± 8.46 | 32.70 ± 11.06 | <0.001 |
| Weighted sleep duration, hours, mean ± SD | 8.11 ± 1.13 | 8.12 ± 1.11 | 8.08 ± 1.50 | 0.677 |
| Social jetlag, hours, mean ± SD | 1.38 ± 1.08 | 1.36 ± 1.06 | 1.70 ± 1.36 | 0.043 |
| Body-image concern score, mean ± SD | 19.16 ± 9.57 | 18.61 ± 9.17 | 27.65 ± 11.47 | <0.001 |
| Social support score, mean ± SD | 59.31 ± 13.33 | 59.75 ± 13.21 | 52.48 ± 13.38 | <0.001 |

Categorical characteristics

| **Characteristic** | **Overall** | **Anxiety-risk negative** | **Anxiety-risk positive** | **P value** |
| --- | --- | --- | --- | --- |
| Female, n (%) | 533 (48.7) | 492 (47.8) | 41 (62.1) | 0.033 |
| Sports major, n (%) | 77 (7.0) | 68 (6.6) | 9 (13.6) | 0.043 |
| Physical inactivity, n (%) | 220 (20.1) | 201 (19.5) | 19 (28.8) | 0.097 |
| High sedentary time, n (%) | 321 (29.3) | 300 (29.2) | 21 (31.8) | 0.748 |
| High smartphone dependency, n (%) | 303 (27.7) | 256 (24.9) | 47 (71.2) | <0.001 |
| Short sleep, n (%) | 129 (14.4) | 120 (14.2) | 9 (19.1) | 0.464 |
| Evening chronotype, n (%) | 654 (59.7) | 610 (59.3) | 44 (66.7) | 0.291 |
| Breakfast skipping, n (%) | 464 (42.4) | 431 (41.9) | 33 (50.0) | 0.244 |
| Current smoking, n (%) | 39 (3.6) | 34 (3.3) | 5 (7.6) | 0.080 |
| Current drinking, n (%) | 215 (19.6) | 198 (19.2) | 17 (25.8) | 0.258 |
| High body-image concern, n (%) | 283 (25.8) | 244 (23.7) | 39 (59.1) | <0.001 |
| Low social support, n (%) | 289 (26.4) | 254 (24.7) | 35 (53.0) | <0.001 |

Note. Anxiety risk was defined as GAD-7 ≥ 10. Values are shown as mean ± SD or n (%). Percentages were calculated using non-missing denominators; denominators may vary across variables because of missing data.

**Supplementary Table S9. Comparison of participants included in and excluded from outcome-specific analyses because of unavailable PHQ-9 or GAD-7 outcome data**

**Part A. Depression-risk outcome availability**

| **Variable** | **Included** | **Excluded** | **P value** |
| --- | --- | --- | --- |
| Age (age_years) | 19.17 ± 1.11 | 19.62 ± 1.87 | 0.010 |
| Sex (sex) | 1.0: 562 (51.3%); 2.0: 534 (48.7%) | 1.0: 55 (44.4%); 2.0: 69 (55.6%) | 0.144 |
| Body mass index (bmi) | 21.97 ± 4.87 | 22.20 ± 4.12 | 0.613 |
| Smartphone dependency score (smartphone_dependency_score) | 24.50 ± 8.88 | 23.80 ± 11.23 | 0.610 |
| Body-image concern score (body_image_concern_score) | 19.16 ± 9.57 | 17.48 ± 5.38 | 0.129 |
| Perceived social support score (social_support_score) | 59.32 ± 13.32 | 50.86 ± 13.98 | 0.012 |

**Part B. Anxiety-risk outcome availability**

| **Variable** | **Included** | **Excluded** | **P value** |
| --- | --- | --- | --- |
| Age (age_years) | 19.17 ± 1.11 | 19.62 ± 1.86 | 0.010 |
| Sex (sex) | 1.0: 562 (51.3%); 2.0: 533 (48.7%) | 1.0: 55 (44.0%); 2.0: 70 (56.0%) | 0.121 |
| Body mass index (bmi) | 21.98 ± 4.87 | 22.15 ± 4.13 | 0.694 |
| Smartphone dependency score (smartphone_dependency_score) | 24.51 ± 8.88 | 23.66 ± 11.21 | 0.533 |
| Body-image concern score (body_image_concern_score) | 19.16 ± 9.57 | 17.75 ± 5.47 | 0.200 |
| Perceived social support score (social_support_score) | 59.31 ± 13.33 | 51.50 ± 13.98 | 0.016 |

Note. This table compares participants included in each outcome-specific analysis with those excluded because the corresponding PHQ-9 or GAD-7 outcome data were unavailable. Percentages were calculated using non-missing denominators. P values were used descriptively to assess potential differences related to outcome availability and were not adjusted for multiple testing.

**Supplementary Table S10. Individual-variable permutation importance for the primary Feature set C random-forest models**

| **Outcome** | **Rank** | **Variable-level predictor** | **Construct** | **Importance** | **SD** | **Directionality summary** |
| --- | --- | --- | --- | --- | --- | --- |
| Anxiety risk | 1 | (high_body_image_concern) | body_image | 0.0055 | 0.0010 | Higher level associated with higher predicted risk |
| Anxiety risk | 2 | (high_smartphone_dependency) | smartphone_dependency | 0.0052 | 0.0011 | Higher level associated with higher predicted risk |
| Anxiety risk | 3 | (social_support_score) | social_support | 0.0045 | 0.0009 | Lower level associated with higher predicted risk |
| Anxiety risk | 4 | (body_image_concern_score) | body_image | 0.0039 | 0.0008 | Higher level associated with higher predicted risk |
| Anxiety risk | 5 | (waist_cm) | anthropometrics | 0.0035 | 0.0006 | Lower level associated with higher predicted risk |
| Anxiety risk | 6 | (weighted_sedentary_hours) | sedentary | 0.0034 | 0.0005 | Nonlinear or unstable trend |
| Anxiety risk | 7 | (low_social_support) | social_support | 0.0030 | 0.0009 | Higher level associated with higher predicted risk |
| Anxiety risk | 8 | (social_jetlag) | social_jetlag | 0.0030 | 0.0007 | Higher level associated with higher predicted risk |
| Anxiety risk | 9 | (monthly_allowance) | socioeconomic | 0.0024 | 0.0005 | Nonlinear or unstable trend |
| Anxiety risk | 10 | (smartphone_dependency_score) | smartphone_dependency | 0.0022 | 0.0007 | Higher level associated with higher predicted risk |
| Anxiety risk | 11 | (weighted_sleep_duration) | sleep | 0.0021 | 0.0003 |  |
| Anxiety risk | 12 | (bmi) | anthropometrics | 0.0021 | 0.0004 |  |
| Anxiety risk | 13 | (evening_type) | chronotype | 0.0017 | 0.0006 |  |
| Anxiety risk | 14 | (grade) | academic_year | 0.0017 | 0.0004 |  |
| Anxiety risk | 15 | (age_years) | anthropometrics | 0.0016 | 0.0002 |  |
| Anxiety risk | 16 | (recreational_screen_time_hours) | recreational_screen_time | 0.0014 | 0.0002 |  |
| Anxiety risk | 17 | (high_unhealthy_diet) | diet | 0.0013 | 0.0004 |  |
| Anxiety risk | 18 | (residence) | residence | 0.0012 | 0.0002 |  |
| Anxiety risk | 19 | (daily_ssb) | diet | 0.0012 | 0.0005 |  |
| Anxiety risk | 20 | (high_recreational_screen) | recreational_screen_time | 0.0011 | 0.0004 |  |
| Depression risk | 1 | (social_support_score) | social_support | 0.0115 | 0.0018 | Lower level associated with higher predicted risk |
| Depression risk | 2 | (breakfast_skipping) | dietary_rhythm | 0.0098 | 0.0014 | Higher level associated with higher predicted risk |
| Depression risk | 3 | (social_jetlag) | social_jetlag | 0.0068 | 0.0010 | Higher level associated with higher predicted risk |
| Depression risk | 4 | (body_image_concern_score) | body_image | 0.0063 | 0.0013 | Higher level associated with higher predicted risk |
| Depression risk | 5 | (high_smartphone_dependency) | smartphone_dependency | 0.0062 | 0.0016 | Higher level associated with higher predicted risk |
| Depression risk | 6 | (smartphone_dependency_score) | smartphone_dependency | 0.0061 | 0.0012 | Higher level associated with higher predicted risk |
| Depression risk | 7 | (high_body_image_concern) | body_image | 0.0060 | 0.0010 | Higher level associated with higher predicted risk |
| Depression risk | 8 | (weighted_sleep_duration) | sleep | 0.0052 | 0.0005 | Lower level associated with higher predicted risk |
| Depression risk | 9 | (waist_cm) | anthropometrics | 0.0044 | 0.0005 | Lower level associated with higher predicted risk |
| Depression risk | 10 | (low_social_support) | social_support | 0.0044 | 0.0008 | Higher level associated with higher predicted risk |
| Depression risk | 11 | (weighted_sedentary_hours) | sedentary | 0.0043 | 0.0004 |  |
| Depression risk | 12 | (bmi) | anthropometrics | 0.0040 | 0.0004 |  |
| Depression risk | 13 | (evening_type) | chronotype | 0.0038 | 0.0006 |  |
| Depression risk | 14 | (recreational_screen_time_hours) | recreational_screen_time | 0.0038 | 0.0004 |  |
| Depression risk | 15 | (residence) | residence | 0.0031 | 0.0003 |  |
| Depression risk | 16 | (number_of_friends) | social_network | 0.0028 | 0.0003 |  |
| Depression risk | 17 | (night_light_exposure_score) | night_light_exposure | 0.0027 | 0.0005 |  |
| Depression risk | 18 | (monthly_allowance) | socioeconomic | 0.0024 | 0.0003 |  |
| Depression risk | 19 | (sex) | sex | 0.0023 | 0.0005 |  |
| Depression risk | 20 | (only_child) | family_structure | 0.0023 | 0.0003 |  |

Note. Permutation importance was calculated as the decrease in AUROC after permutation. The Construct column identifies the prespecified construct assigned to each variable; related variables were grouped for the construct-level ranking in Figure 4.

**Supplementary Table S11. Feature set C-clean sensitivity analysis after removing duplicated dichotomized high-risk indicators**

**Part A. Internal validation**

| **Metric** | **Depression risk** | **Anxiety risk** |
| --- | --- | --- |
| AUROC | 0.771 | 0.788 |
| PR-AUC | 0.277 | 0.257 |
| Brier score | 0.094 | 0.064 |
| Default recall | 0.111 | 0.034 |
| Default specificity | 0.976 | 0.995 |
| Youden threshold | 0.261 | 0.239 |
| Youden recall | 0.704 | 0.642 |
| Youden specificity | 0.719 | 0.813 |

**Part B. External validation using sleep-time preprocessing correction**

| **Metric** | **Depression risk** | **Anxiety risk** |
| --- | --- | --- |
| AUROC | 0.820 | 0.807 |
| PR-AUC | 0.407 | 0.214 |
| Brier score | 0.090 | 0.062 |
| Baseline Brier score | 0.091 | 0.051 |
| Calibration intercept | -0.287 | -0.669 |
| Calibration slope | 1.824 | 1.600 |
| Default recall | 0.189 | 0.038 |
| Default specificity | 0.984 | 0.993 |
| Youden recall | 0.750 | 0.658 |
| Youden specificity | 0.723 | 0.788 |

**Part C. Reciprocal validation using sleep-time preprocessing correction**

| **Outcome** | **AUROC** | **PR-AUC** | **Brier score** | **Default recall** | **Default specificity** |
| --- | --- | --- | --- | --- | --- |
| Depression risk | 0.774 | 0.307 | 0.093 | 0.245 | 0.964 |
| Anxiety risk | 0.801 | 0.252 | 0.061 | 0.045 | 0.999 |

Note. Feature set C-clean removed high_smartphone_dependency, high_body_image_concern, low_social_support, high_unhealthy_diet, high_night_light_exposure, and high_recreational_screen. Short_sleep and high_social_jetlag were retained as threshold indicators.

**Supplementary Table S12. Reciprocal validation sensitivity analysis**

| **Analysis direction** | **Outcome** | **Development n** | **Development positive n** | **External validation n** | **External validation positive n** | **AUROC** | **PR-AUC** | **Brier score** |
| --- | --- | --- | --- | --- | --- | --- | --- | --- |
| Shanghai development after sleep-time preprocessing correction -> original dataset external validation | Depression risk | 1468 | 148 | 1096 | 102 | 0.760 | 0.299 | 0.076 |
| Shanghai development after sleep-time preprocessing correction -> original dataset external validation | Anxiety risk | 1468 | 79 | 1095 | 66 | 0.823 | 0.272 | 0.050 |

Note. In this reciprocal validation sensitivity analysis, the independent university dataset was used as the development sample after the sleep-time preprocessing correction, and the original development dataset was used as the external validation sample. Feature engineering, preprocessing, and Random Forest hyperparameters followed the primary analysis. Cutoffs for distribution-based binary indicators were derived from the reciprocal development sample and then fixed when applied to the reciprocal external validation sample. PR-AUC was calculated as average precision.

**Supplementary Table S13. Exploratory overlap between depression-risk and anxiety-risk outcomes**

**Part A. Development sample outcome overlap**

| **Measure** | **Result** |
| --- | --- |
| Both depression/anxiety labels available | n = 1095 |
| Neither depression nor anxiety risk | 975 (89.0%) |
| Depression risk only | 54 (4.9%) |
| Anxiety risk only | 18 (1.6%) |
| Both depression and anxiety risk | 48 (4.4%) |
| Odds ratio | 48.15 (95% CI 26.24–88.35) |
| Phi | 0.553 |
| Cramer's V | 0.553 |
| Cohen's kappa | 0.538 |
| P value | <0.001 |

**Part B. External validation sample outcome overlap**

| **Measure** | **Result** |
| --- | --- |
| External validation sample | n = 1468 |
| Neither depression nor anxiety risk | 1304 (88.8%) |
| Depression risk only | 85 (5.8%) |
| Anxiety risk only | 16 (1.1%) |
| Both depression and anxiety risk | 63 (4.3%) |
| Odds ratio | 60.41 (95% CI 33.45–109.07) |
| Phi | 0.552 |
| Cramer's V | 0.552 |
| Cohen's kappa | 0.521 |
| P value | <0.001 |

**Part C. Exploratory overlap in model predictions and top constructs**

| **Domain** | **Measure** | **Result** |
| --- | --- | --- |
| External PHQ-9/GAD-7 total-score correlation | Pearson correlation | r = 0.823 (95% CI 0.806–0.839) |
| External PHQ-9/GAD-7 total-score correlation | Spearman correlation | rho = 0.800 |
| External predicted probability correlation | Pearson correlation | r = 0.915 (95% CI 0.906–0.923) |
| External predicted probability correlation | Spearman correlation | rho = 0.875 |
| Discordant high-risk predictions, default threshold | Depression-only; anxiety-only; both | 45; 7; 54 |
| Discordant high-risk predictions, fixed Youden threshold | Depression-only; anxiety-only; both | 246; 5; 282 |
| Top construct overlap | Shared constructs | 8/12 |
| Top construct overlap | Jaccard similarity | 0.667 |
| Top construct overlap | Shared constructs | Perceived social support; Body-image concern; Smartphone dependency; Social jetlag; Sleep duration; Waist circumference; Weighted average sedentary time; Body mass index |
| Top construct overlap | Depression-specific constructs | Breakfast skipping; Evening chronotype |
| Top construct overlap | Anxiety-specific constructs | Recreational screen time; Monthly living allowance |

Note. This descriptive analysis examined overlap and discordance between depression-risk and anxiety-risk outcomes, defined as PHQ-9 and GAD-7 scores of 10 or higher, respectively.

**Supplementary Table S14a. External validation discrimination and calibration metrics**

| **Metric** | **Depression risk** | **Anxiety risk** |
| --- | --- | --- |
| n | 1468 | 1468 |
| Positive n (%) | 148 (10.1%) | 79 (5.4%) |
| AUROC (95% CI) | 0.813 (0.776–0.848) | 0.809 (0.765–0.851) |
| PR-AUC (95% CI) | 0.402 (0.325–0.491) | 0.205 (0.144–0.300) |
| Brier score (95% CI) | 0.090 (0.083–0.098) | 0.063 (0.056–0.070) |
| Prevalence-based baseline Brier score (95% CI) | 0.091 (0.078–0.103) | 0.051 (0.040–0.062) |
| Calibration intercept (95% CI) | -0.668 (-0.920 to -0.413) | -1.227 (-1.571 to -0.903) |
| Calibration slope (95% CI) | 1.401 (1.196–1.651) | 1.102 (0.902–1.348) |

Note. PR-AUC was calculated as average precision. Confidence intervals were estimated using ordinary participant-level bootstrap resampling of the external validation predictions with 2,000 replicates. Calibration intercept and slope were estimated using logistic calibration models based on the logit of predicted probabilities.

**Supplementary Table S14b. External validation threshold-dependent metrics**

**Depression risk**

| **Metric** | **Default threshold = 0.5** | **Fixed internal Youden threshold = 0.226** |
| --- | --- | --- |
| Recall (95% CI) | 0.318 (0.243–0.392) | 0.791 (0.725–0.855) |
| Specificity (95% CI) | 0.961 (0.950–0.971) | 0.689 (0.664–0.713) |
| Precision (95% CI) | 0.475 (0.376–0.575) | 0.222 (0.187–0.258) |
| F1 score (95% CI) | 0.381 (0.302–0.455) | 0.346 (0.300–0.394) |
| Balanced accuracy (95% CI) | 0.639 (0.602–0.677) | 0.740 (0.705–0.774) |

**Anxiety risk**

| **Metric** | **Default threshold = 0.5** | **Fixed internal Youden threshold = 0.251** |
| --- | --- | --- |
| Recall (95% CI) | 0.241 (0.148–0.342) | 0.608 (0.506–0.711) |
| Specificity (95% CI) | 0.970 (0.961–0.978) | 0.828 (0.807–0.848) |
| Precision (95% CI) | 0.311 (0.197–0.435) | 0.167 (0.125–0.215) |
| F1 score (95% CI) | 0.271 (0.173–0.371) | 0.262 (0.203–0.371) |
| Balanced accuracy (95% CI) | 0.605 (0.559–0.656) | 0.718 (0.664–0.771) |

**Point confusion matrices**

| **Outcome** | **Threshold** | **TP** | **FP** | **TN** | **FN** |
| --- | --- | --- | --- | --- | --- |
| Depression risk | Default threshold = 0.5 | 47 | 52 | 1268 | 101 |
| Depression risk | Fixed internal Youden threshold = 0.226 | 117 | 411 | 909 | 31 |
| Anxiety risk | Default threshold = 0.5 | 19 | 42 | 1347 | 60 |
| Anxiety risk | Fixed internal Youden threshold = 0.251 | 48 | 239 | 1150 | 31 |

Note. Confidence intervals were estimated using ordinary participant-level bootstrap resampling of the external validation predictions with 2,000 replicates. Stratified participant-level bootstrap was prespecified as a secondary sensitivity analysis and is not shown in this table. TP, FP, TN, and FN are point counts from the complete external validation dataset.

**Supplementary Figure S1. Analysis workflow**

Overview of the internal model-development workflow, including feature set definitions, repeated stratified 5-fold cross-validation with 5 repeats, pipeline preprocessing, internal validation, calibration and threshold analyses, model interpretation, and sensitivity analyses. External validation was conducted separately using the saved Feature set C Random Forest pipelines and the fixed Feature set D sensitivity pipeline.

**Supplementary Table S15a. Legacy development-only optimized random-forest performance**

| **Outcome** | **Analysis** | **n** | **Positive n** | **Predictor count** | **AUROC** | **PR-AUC** | **Brier score** |
| --- | --- | --- | --- | --- | --- | --- | --- |
| Depression risk | Legacy nested optimization | 1096 | 102 | Superseded | Not retained | Not retained | Not retained |
| Anxiety risk | Legacy nested optimization | 1095 | 66 | Superseded | Not retained | Not retained | Not retained |

Note. Default RF is the prespecified full Feature set C pipeline evaluated within the 10-fold feature-selection workflow; these estimates are distinct from the primary repeated stratified 5-fold cross-validation with five repeats. Optimized RF is the development-only AUROC-optimized pipeline.

**Supplementary Table S15b. Calibration and threshold-dependent performance of the legacy development-only optimized random-forest models**

| **Outcome** | **Analysis** | **Calibration intercept** | **Calibration slope** | **Default recall / specificity** | **Youden threshold** | **Youden recall / specificity** |
| --- | --- | --- | --- | --- | --- | --- |
| Depression risk | Legacy nested optimization | Not retained | Not retained | Not retained | Not retained | Not retained |
| Anxiety risk | Legacy nested optimization | Not retained | Not retained | Not retained | Not retained | Not retained |

Note. The development-derived threshold was estimated from nested cross-fitted development predictions and was not adjusted in Shanghai. Default threshold = 0.5.

**Supplementary Table S16a. Predictors selected by the archived development-only feature-selection procedure**

| **Outcome** | **Predictor count** | **Predictors in locked input order** |
| --- | --- | --- |
| Depression risk | 12 | high_smartphone_dependency, smartphone_dependency_score, high_body_image_concern, body_image_concern_score, social_support_score, low_social_support, binge_drinking, monthly_allowance, recreational_screen_time_hours, student_organization_participation, social_jetlag, grade |
| Anxiety risk | 15 | high_smartphone_dependency, low_social_support, smartphone_dependency_score, high_body_image_concern, social_support_score, high_unhealthy_diet, only_child, weighted_sedentary_hours, body_image_concern_score, sex, breakfast_skipping, recreational_screen_time_hours, unhealthy_diet_score, age_years, waist_cm |

Note. Final rankings were calculated in the full development cohort after cross-validated predictor-count selection. External validation data were not used.

**Supplementary Table S16b. Default and legacy development-only optimized random-forest specifications**

| **Outcome** | **Trees** | **Max depth** | **Max features** | **Min leaf** | **Min split** | **Class weight** | **Max samples** | **Calibration** | **Development Youden threshold** |
| --- | --- | --- | --- | --- | --- | --- | --- | --- | --- |
| Depression risk | 500 | 4 | 0.3 | 5 | 2 | balanced_subsample | None | sigmoid | 0.086 |
| Anxiety risk | 1200 | 8 | sqrt | 5 | 20 | balanced | 0.85 | sigmoid | 0.083 |

Note. Randomized search used AUROC as the optimization score and was conducted exclusively within development data. PR-AUC and Brier score were evaluated as secondary performance measures. Calibration intercept and slope were estimated from cross-fitted predictions, and the calibration method was selected within development data using Brier score.

**Supplementary Table S17. Legacy development-only feature-selection performance by predictor number**

| **Outcome** | **Number of ranked predictors** | **AUROC** | **PR-AUC** | **Brier score** | **Calibration slope** |
| --- | --- | --- | --- | --- | --- |
| Depression risk | Legacy 10-fold artifact | Not retained | Not retained | Not retained | Not retained |
| Anxiety risk | Legacy 10-fold artifact | Not retained | Not retained | Not retained | Not retained |

Note. Predictor ranking was recalculated from training data in each stratified 10-fold split. The selected count was the smallest candidate within one standard error of the highest mean fold AUROC. Confidence intervals were estimated by 2000 participant-level stratified bootstrap resamples of out-of-fold predictions.

**Supplementary Table S18. Predictive values under assumed outcome prevalence levels**

| **Outcome** | **Assumed prevalence** | **Sensitivity** | **Specificity** | **PPV** | **NPV** |
| --- | --- | --- | --- | --- | --- |
| Depression risk | 5% | 0.791 | 0.689 | 0.118 | 0.984 |
| Depression risk | 10% | 0.791 | 0.689 | 0.220 | 0.967 |
| Depression risk | 15% | 0.791 | 0.689 | 0.309 | 0.949 |
| Depression risk | 20% | 0.791 | 0.689 | 0.388 | 0.929 |
| Anxiety risk | 5% | 0.608 | 0.828 | 0.157 | 0.976 |
| Anxiety risk | 10% | 0.608 | 0.828 | 0.282 | 0.950 |
| Anxiety risk | 15% | 0.608 | 0.828 | 0.384 | 0.923 |
| Anxiety risk | 20% | 0.608 | 0.828 | 0.469 | 0.894 |

Note. Sensitivity and specificity were taken from the fixed development-derived Youden threshold applied to the locked primary Feature set C external predictions. PPV and NPV were calculated under each assumed prevalence using standard predictive-value formulas; the model and threshold were not changed.

**Supplementary Table S19. Development-versus-external-validation case-mix comparison.**

| **Variable** | **Development (n=1,267)** | **External (n=1,468)** | **Development missing %** | **External missing %** | **SMD** |
| --- | --- | --- | --- | --- | --- |
| age_years | 19.21 ± 1.21 | 19.76 ± 1.51 | 4.1831 | 0.545 | -0.3981 |
| sex | 1: 617 (50.6%); 2: 603 (49.4%) | 1: 789 (53.7%); 2: 679 (46.3%) | 3.7096 | 0.0 |  |
| grade | 1: 1 (0.1%); 2: 799 (63.3%); 3: 389 (30.8%); 4: 60 (4.8%); 5: 11 (0.9%); 6: 2 (0.2%) | 2: 536 (36.5%); 3: 312 (21.3%); 4: 357 (24.3%); 5: 261 (17.8%); 6: 2 (0.1%) | 0.3946 | 0.0 |  |
| sports_major | 0: 1163 (92.2%); 1: 99 (7.8%) | 0: 1468 (100.0%) | 0.3946 | 0.0 | 0.4126 |
| bmi | 21.99 ± 4.81 | 22.22 ± 4.26 | 5.7616 | 0.4087 | -0.0494 |
| waist_cm | 76.74 ± 11.37 | 78.13 ± 11.19 | 6.3141 | 0.0 | -0.1236 |
| weighted_sedentary_hours | 5.79 ± 3.32 | 5.76 ± 2.56 | 9.3923 | 0.0 | 0.0095 |
| weighted_sleep_duration | 8.12 ± 1.13 | 8.30 ± 0.93 | 27.4665 | 0.4087 | -0.175 |
| recreational_screen_time_hours | 4.06 ± 3.25 | 3.76 ± 2.47 | 8.2084 | 0.0 | 0.1027 |
| social_jetlag | 1.38 ± 1.07 | 1.28 ± 0.86 | 27.4665 | 0.4087 | 0.0964 |
| smartphone_dependency_score | 24.46 ± 9.04 | 24.38 ± 8.99 | 7.9716 | 0.0 | 0.0088 |
| body_image_concern_score | 19.12 ± 9.49 | 18.32 ± 9.31 | 11.3654 | 0.0 | 0.0856 |
| social_support_score | 59.16 ± 13.38 | 60.50 ± 13.94 | 11.839 | 0.0 | -0.0986 |
| high_sedentary | 0: 816 (71.1%); 1: 332 (28.9%) | 0: 989 (67.4%); 1: 479 (32.6%) | 9.3923 | 0.0 | -0.0804 |
| short_sleep | 0: 787 (85.6%); 1: 132 (14.4%) | 0: 1339 (91.6%); 1: 123 (8.4%) | 27.4665 | 0.4087 | 0.1881 |
| high_smartphone_dependency | 0: 848 (72.7%); 1: 318 (27.3%) | 0: 1059 (72.1%); 1: 409 (27.9%) | 7.9716 | 0.0 | -0.0132 |
| high_body_image_concern | 0: 836 (74.4%); 1: 287 (25.6%) | 0: 1131 (77.0%); 1: 337 (23.0%) | 11.3654 | 0.0 | 0.0607 |
| low_social_support | 0: 819 (73.3%); 1: 298 (26.7%) | 0: 1127 (76.8%); 1: 341 (23.2%) | 11.839 | 0.0 | 0.0798 |
| high_social_jetlag | 0: 740 (80.5%); 1: 179 (19.5%) | 0: 1190 (81.4%); 1: 272 (18.6%) | 27.4665 | 0.4087 | 0.0222 |
| breakfast_skipping | 0: 649 (57.7%); 1: 475 (42.3%) | 0: 840 (57.2%); 1: 628 (42.8%) | 11.2865 | 0.0 | -0.0105 |
| late_dinner | 0: 1007 (89.6%); 1: 117 (10.4%) | 0: 1271 (86.6%); 1: 197 (13.4%) | 11.2865 | 0.0 | -0.093 |
| daily_ssb | 0: 437 (37.9%); 1: 716 (62.1%) | 0: 527 (35.9%); 1: 941 (64.1%) | 8.9976 | 0.0 | -0.0415 |
| high_unhealthy_diet | 0: 718 (62.1%); 1: 439 (37.9%) | 0: 820 (55.9%); 1: 648 (44.1%) | 8.6819 | 0.0 | -0.1263 |
| depression_risk prevalence | 102/1096 (9.3%) | 148/1468 (10.1%) | 13.4964 | 0.0 |  |
| anxiety_risk prevalence | 66/1095 (6.0%) | 79/1468 (5.4%) | 13.5754 | 0.0 |  |

Note. Continuous variables are summarized as mean +/- SD and categorical variables as n (%). SMD, standardized mean difference. Values are descriptive and are not used to retune the locked models.

**Supplementary Table S20. Corrected primary Feature set C internal-validation analysis with fold-specific percentile cutoffs.**

| **Metric** | **Depression risk** | **Anxiety risk** |
| --- | --- | --- |
| Participants, n | 1096 | 1095 |
| Positive cases, n | 102 | 66 |
| Prevalence | 0.0931 | 0.0603 |
| AUROC | 0.7731 | 0.7957 |
| PR-AUC / average precision | 0.2660 | 0.2656 |
| Brier score | 0.0911 | 0.0617 |
| Calibration intercept | -0.8915 | -0.9611 |
| Calibration slope | 1.1192 | 1.1295 |
| Recall at threshold 0.5 | 0.1765 | 0.1667 |
| Specificity at threshold 0.5 | 0.9628 | 0.9845 |

Both rows describe Feature set C. Percentile cutoffs were estimated within each training fold; fold_specific_percentile_cutoffs = True for both outcomes.

Note. These corrected estimates are the formal internal-validation results and use training-fold preprocessing before application to held-out folds.

**Supplementary Table S21. Descriptive external group-permutation importance for locked Feature set C models.**

**Depression risk**

| **Construct** | **Mean AUROC decrease** | **SD** | **2.5th percentile** | **97.5th percentile** |
| --- | --- | --- | --- | --- |
| social support | 0.0376 | 0.0064 | 0.0266 | 0.0490 |
| smartphone dependency | 0.0700 | 0.0132 | 0.0462 | 0.0912 |
| body image | 0.0433 | 0.0065 | 0.0302 | 0.0553 |
| sleep | 0.0059 | 0.0022 | 0.0009 | 0.0092 |
| social jetlag | 0.0007 | 0.0031 | -0.0057 | 0.0059 |
| diet | -0.0013 | 0.0023 | -0.0057 | 0.0033 |
| sedentary | 0.0007 | 0.0014 | -0.0019 | 0.0031 |
| anthropometrics | 0.0041 | 0.0029 | -0.0018 | 0.0082 |

Locked baseline AUROC = 0.8132; 100 joint permutations per construct. Status for all rows: descriptive external group permutation.

**Anxiety risk**

| **Construct** | **Mean AUROC decrease** | **SD** | **2.5th percentile** | **97.5th percentile** |
| --- | --- | --- | --- | --- |
| social support | 0.0310 | 0.0087 | 0.0111 | 0.0460 |
| smartphone dependency | 0.0804 | 0.0150 | 0.0509 | 0.1100 |
| body image | 0.0551 | 0.0099 | 0.0336 | 0.0727 |
| sleep | -0.0003 | 0.0017 | -0.0036 | 0.0025 |
| social jetlag | 0.0045 | 0.0040 | -0.0029 | 0.0115 |
| diet | -0.0002 | 0.0034 | -0.0067 | 0.0056 |
| sedentary | -0.0007 | 0.0027 | -0.0062 | 0.0035 |
| anthropometrics | 0.0096 | 0.0053 | -0.0002 | 0.0173 |

Locked baseline AUROC = 0.8088; 100 joint permutations per construct. Status for all rows: descriptive external group permutation.

| **Construct** | **Variables jointly permuted** |
| --- | --- |
| social support | social_support_score + low_social_support |
| smartphone dependency | smartphone_dependency_score + high_smartphone_dependency |
| body image | body_image_concern_score + high_body_image_concern |
| sleep | weighted_sleep_duration + short_sleep |
| social jetlag | social_jetlag + high_social_jetlag |
| diet | unhealthy_diet_score + high_unhealthy_diet |
| sedentary | weighted_sedentary_hours + high_sedentary |
| anthropometrics | age_years + bmi + waist_cm |

Note. Related variables were jointly permuted within prespecified constructs on the locked external predictions. Group importance was defined as the baseline AUROC minus the AUROC after joint permutation. Values are descriptive, exploratory, and not used for model selection.

**Supplementary Table S22. Unweighted and class-weighted logistic-regression comparator analysis**

| **Metric** | **Depression unweighted** | **Depression balanced** | **Anxiety unweighted** | **Anxiety balanced** |
| --- | --- | --- | --- | --- |
| n | 1096 | 1096 | 1095 | 1095 |
| positive_n | 102 | 102 | 66 | 66 |
| prevalence | 0.0931 | 0.0931 | 0.0603 | 0.0603 |
| AUROC | 0.7470 | 0.7314 | 0.7924 | 0.7871 |
| PR_AUC | 0.2425 | 0.2516 | 0.2014 | 0.1999 |
| Brier | 0.0809 | 0.1782 | 0.0557 | 0.1457 |
| calibration_intercept | -0.7367 | -2.0774 | -0.9226 | -2.3792 |
| calibration_slope | 0.6161 | 0.5235 | 0.5778 | 0.4407 |

Note. Logistic regression was evaluated as a comparator using the same development Feature set C and repeated cross-validation. The random-forest primary model was unchanged. The S4 algorithm-comparison script used L2 regularization with C = 1e6, solver = lbfgs, max_iter = 3000, and class_weight = balanced, whereas this separate unweighted-versus-class-weighted comparison used the default C = 1.0 with the same solver and max_iter. The estimates are therefore not interchangeable.

**Supplementary Table S23. Development-only predictor selection frequency across outer folds.**

| **Outcome** | **Predictor** | **Eligible folds** | **Selected/top-ranked folds** | **Selection frequency (%)** |
| --- | --- | --- | --- | --- |
| depression | smartphone_dependency_score | 25 | 25 | 100.0 |
| depression | high_smartphone_dependency | 25 | 22 | 88.0 |
| depression | body_image_concern_score | 25 | 16 | 64.0 |
| depression | social_support_score | 25 | 15 | 60.0 |
| depression | high_body_image_concern | 25 | 13 | 52.0 |
| depression | binge_drinking | 25 | 13 | 52.0 |
| depression | low_social_support | 25 | 12 | 48.0 |
| depression | high_unhealthy_diet | 25 | 6 | 24.0 |
| depression | night_light_exposure_score | 25 | 6 | 24.0 |
| depression | social_jetlag | 25 | 4 | 16.0 |
| depression | residence | 25 | 4 | 16.0 |
| depression | number_of_friends | 25 | 3 | 12.0 |
| depression | parental_education_high | 25 | 3 | 12.0 |
| depression | monthly_allowance | 25 | 3 | 12.0 |
| depression | only_child | 25 | 3 | 12.0 |
| depression | waist_cm | 25 | 2 | 8.0 |
| depression | bmi | 25 | 2 | 8.0 |
| depression | current_smoking | 25 | 2 | 8.0 |
| depression | current_drinking | 25 | 2 | 8.0 |
| depression | evening_type | 25 | 2 | 8.0 |
| depression | age_years | 25 | 2 | 8.0 |
| depression | sex | 25 | 2 | 8.0 |
| depression | late_dinner | 25 | 2 | 8.0 |
| depression | short_sleep | 25 | 2 | 8.0 |
| depression | recreational_screen_time_hours | 25 | 2 | 8.0 |
| depression | breakfast_skipping | 25 | 2 | 8.0 |
| depression | high_sedentary | 25 | 2 | 8.0 |
| depression | grade | 25 | 2 | 8.0 |
| depression | sports_major | 25 | 1 | 4.0 |
| depression | weighted_sleep_duration | 25 | 1 | 4.0 |
| depression | student_organization_participation | 25 | 1 | 4.0 |
| depression | physical_inactivity | 25 | 1 | 4.0 |
| depression | high_social_jetlag | 25 | 1 | 4.0 |
| depression | unhealthy_diet_score | 25 | 1 | 4.0 |
| anxiety | low_social_support | 25 | 24 | 96.0 |
| anxiety | high_smartphone_dependency | 25 | 23 | 92.0 |
| anxiety | body_image_concern_score | 25 | 20 | 80.0 |
| anxiety | social_support_score | 25 | 20 | 80.0 |
| anxiety | smartphone_dependency_score | 25 | 19 | 76.0 |
| anxiety | high_body_image_concern | 25 | 16 | 64.0 |
| anxiety | weighted_sedentary_hours | 25 | 12 | 48.0 |
| anxiety | waist_cm | 25 | 10 | 40.0 |
| anxiety | sex | 25 | 9 | 36.0 |
| anxiety | age_years | 25 | 5 | 20.0 |
| anxiety | breakfast_skipping | 25 | 5 | 20.0 |
| anxiety | monthly_allowance | 25 | 5 | 20.0 |
| anxiety | residence | 25 | 5 | 20.0 |
| anxiety | high_unhealthy_diet | 25 | 4 | 16.0 |
| anxiety | recreational_screen_time_hours | 25 | 4 | 16.0 |
| anxiety | late_dinner | 25 | 4 | 16.0 |
| anxiety | high_sedentary | 25 | 4 | 16.0 |
| anxiety | only_child | 25 | 3 | 12.0 |
| anxiety | unhealthy_diet_score | 25 | 2 | 8.0 |
| anxiety | night_light_exposure_score | 25 | 2 | 8.0 |
| anxiety | social_jetlag | 25 | 2 | 8.0 |
| anxiety | number_of_friends | 25 | 2 | 8.0 |
| anxiety | student_organization_participation | 25 | 2 | 8.0 |
| anxiety | high_recreational_screen | 25 | 2 | 8.0 |
| anxiety | weighted_sleep_duration | 25 | 1 | 4.0 |
| anxiety | physical_inactivity | 25 | 1 | 4.0 |
| anxiety | bmi | 25 | 1 | 4.0 |
| anxiety | sports_major | 25 | 1 | 4.0 |
| anxiety | binge_drinking | 25 | 1 | 4.0 |
| anxiety | parental_education_high | 25 | 1 | 4.0 |
| anxiety | current_smoking | 25 | 1 | 4.0 |
| anxiety | current_drinking | 25 | 1 | 4.0 |

Note. Frequencies were calculated across the 25 outer folds of the retained development-only nested feature-selection analysis. These selection-frequency summaries are descriptive traceability outputs from the archived development-only selection pathway and are not treated as formal leakage-free model-selection estimates. External data were not used for selection or ranking.

**Supplementary Table S24. Prediction-only sensitivity to the invariant Shanghai sports-major coding**

| **Metric** | **Depression risk current 0** | **Depression risk counterfactual 1** | **Anxiety risk current 0** | **Anxiety risk counterfactual 1** |
| --- | --- | --- | --- | --- |
| n | 1468 | 1468 | 1468 | 1468 |
| Positive n | 148 | 148 | 79 | 79 |
| AUROC | 0.813 | 0.814 | 0.809 | 0.800 |
| PR-AUC | 0.402 | 0.401 | 0.205 | 0.207 |
| Brier score | 0.090 | 0.095 | 0.063 | 0.070 |
| Calibration intercept | -0.668 | -0.686 | -1.227 | -1.258 |
| Calibration slope | 1.401 | 1.596 | 1.102 | 1.317 |

Note. The analysis applied the saved locked primary Feature set C random-forest pipelines to the sleep-fix external feature matrix after setting sports_major to 0 for all records (confirmed current mapping) or 1 for all records (counterfactual mapping). The Shanghai sport code was 2 for all retained records and denoted non-sports majors. No model was refitted, recalibrated, or threshold-adjusted. The counterfactual is not an alternative external-validation estimate; it quantifies sensitivity to the invariant field coding.

**Supplementary Table S25. Feature set D external validation performance.**

| **Metric** | **Depression risk** | **Anxiety risk** |
| --- | --- | --- |
| n | 1,468 | 1,468 |
| Positive n (%) | 148 (10.1%) | 79 (5.4%) |
| AUROC (95% CI) | 0.845 (0.812 to 0.874) | 0.854 (0.810 to 0.891) |
| PR-AUC (95% CI) | 0.465 (0.382 to 0.545) | 0.278 (0.196 to 0.389) |
| Brier score (95% CI) | 0.080 (0.073 to 0.087) | 0.053 (0.047 to 0.060) |
| Calibration intercept (95% CI) | -0.034 (-0.337 to 0.270) | -0.486 (-0.906 to -0.075) |
| Calibration slope (95% CI) | 1.771 (1.522 to 2.058) | 1.554 (1.263 to 1.898) |

Note. Feature set D comprised 70 variables and used the fixed sensitivity-analysis Random Forest specification (n_estimators = 300, min_samples_leaf = 5, class_weight = balanced, random_state = 2026), with numeric median imputation and standard scaling and categorical most-frequent imputation with one-hot encoding. One outcome-specific pipeline was refit for each outcome on the full development sample and applied to the Shanghai external cohort. The Shanghai sport code was 2 for all retained records and denoted non-sports majors; it was mapped to sports_major = 0. No feature selection, hyperparameter tuning, recalibration, or threshold selection used the Shanghai outcomes. Confidence intervals were estimated using 2,000 participant-level ordinary bootstrap replicates. These results are an external-validation sensitivity analysis and do not replace the primary Feature set C estimates.

**Supplementary Figure S2. Incremental predictor-selection curves for the legacy development-only optimization pathway. AUROC and 95% confidence intervals are shown as the number of training-fold-ranked Feature set C predictors increased. Dashed vertical lines indicate the count selected by the one-standard-error rule. The Selected: 10 labels refer to the separate AUROC-based 10-fold historical analysis; the 12/15 predictors in Supplementary Table S16a came from the historical development-locking procedure using five-fold inner-CV average precision.**

**Supplementary Figure S3. External decision-curve analysis for the legacy development-only optimized random-forest models. Net benefit was evaluated at threshold probabilities from 0.05 to 0.30 after the models had been locked in development data. Shaded bands show 95% bootstrap confidence intervals.**

**Supplementary Figure S4. External calibration curves for the legacy development-only optimized random-forest models. Dashed diagonals indicate perfect calibration and horizontal dotted lines indicate observed outcome prevalence.**

**Supplementary Figure S5. External decision-curve analysis for the locked primary Feature set C random-forest models. Curves show net benefit across threshold probabilities from 0.05 to 0.30 for depression-risk and anxiety-risk outcomes, compared with treat-all and treat-none strategies. Shaded areas show 95% ordinary participant-level bootstrap intervals based on 2,000 replicates. The external cohort was not used for feature selection, hyperparameter tuning, recalibration, or threshold selection.**
